# Heavy metal levels and flavonoid intakes are associated with chronic obstructive pulmonary disease: an NHANES analysis (2007–2010 to 2017–2018)

**DOI:** 10.1186/s12889-023-17250-x

**Published:** 2023-11-24

**Authors:** Zhaoqi Yan, Yifeng Xu, Keke Li, Liangji Liu

**Affiliations:** 1 Jiangxi University of Traditional Chinese Medicine, Graduate school, Yangming Road, Nanchang, Jiangxi China; 2 Department of Respiratory and Critical Care Medicine, Hospital of Jiangxi University of Traditional Chinese Medicine, 445 Bayi Dadao, Nanchang, Jiangxi China

**Keywords:** Anthocyanidins, Chronic Obstructive Pulmonary Disease, Cadmium, Lead, Dietary health, Flavonoids, Metal exposure, National Health and Nutrition Examination Survey

## Abstract

**Background:**

The association between exposure to environmental metals and chronic obstructive pulmonary disease (COPD) is preventing chronic lung diseases. However, little is currently known about the interaction between heavy metals and flavonoids in relation to the risk of COPD. This study aims to bridge this knowledge gap by leveraging The National Health and Nutrition Examination Survey (NHANES) database to evaluate thecorrelation between blood levels of heavy metals (cadmium, lead, mercury) and the intake of various flavonoid compounds (isoflavones, anthocyanidins, flavan-3-ols, flavanones, flavones, flavonols, total flavonoids). Additionally, appropriate dietary recommendations are provided based on the study findings.

**Materials and methods:**

Cross-sectional analysis was conducted using the 2007–2010 and 2017–2018 NHANES data. Specialized weighted complex survey design analysis software was used for data analysis. Multivariate logistic regression models and restricted cubic splines (RCS) were used to evaluate the relationship between blood heavy metal levels, flavonoids intake, and COPD incidence in all participants, and to explore the effect of different levels of flavonoids intake on COPD caused by heavy metal exposure.

**Results:**

A total of 7,265 adults aged ≥ 40 years were analyzed. Higher levels of blood cadmium (Cd), blood lead and Anthocyanidin (AC) intake were independently associated with an increased risk of COPD (Cd highest quantile vs. lowest: OR = 1.73, 95% CI, 1.25–2.3; Lead highest quantile vs. lowest quantile: OR = 1.44, 95% CI, 1.11–1.86; AC highest quantile vs. lowest: OR = 0.73, 95% CI, 0.54–0.99). When AC intake exceeded 11.56 mg/d, the effect of Cd exposure on COPD incidence decreased by 27%, and this finding was more significant in smokers.

**Conclusion:**

Higher levels of Cd (≥ 0.45ug/L) and lead (≥ 0.172 ug/L) were positively correlated with the risk of COPD among participants aged 40 years and above, while AC intake (≥ 11.56 mg/d) could reduce the risk related to blood Cd.

**Supplementary Information:**

The online version contains supplementary material available at 10.1186/s12889-023-17250-x.

## Introduction

 Chronic obstructive pulmonary disease (COPD) is an inflammatory disease that obstructs lung airflow, leading to breathing difficulties [1]. COPD has become the third leading cause of death worldwide, imposing a significant economic burden, particularly in low- and middle-income countries (LMIC), where the mortality rate exceeds 80% [2, 3]. In addition to smoking, environmental and occupational factors such as exposure to smoke from biomass fuel combustion and high levels of air pollution should not be overlooked in the development of COPD. It is now understood that COPD primarily results from long-term airway inflammation and oxidative stress, leading to pulmonary alveolar destruction and increased activity of elastolytic enzymes. Consequently, airway remodeling occurs, leading to irreversible lung function impairment [4].

The past decade has witnessed significant industrial advances leading to an increase in the proportion of the global disease burden attributed to environmental factors, reaching 24%,which is reportedly even higher in developing countries [5], heavy metal pollution accounts for a substantial proportion. Heavy metals are metals with a density greater than 5 g·cm^−3^, with have significant biological toxicity and are widely distributed in the environment, including soil, water, air, dust, the human food chain, and manufactured products. Smoking and individuals working in heavy industries are vulnerable to heavy metal exposure [6], and these factors are closely associated with chronic airway inflammation and oxidative stress. These harmful heavy metals adversely affect antioxidant enzymes, especially superoxide dismutase and catalase, hastening the deterioration of collagen and elastin proteins. This process damages the lung’s cellular barrier function, leading to emphysema [7, 8]. Simultaneously, heavy metals also have the ability to induce reactive oxygen species by stimulating polymorphonuclear leukocytes and phagocytes, triggering chronic inflammatory responses that harm lung tissues. Over time, these damages contribute to the development of COPD. Currently, traditional chelating agents used to mitigate heavy metal toxicity still face certain issues. These issues manifest as unavoidable neurotoxicity and the loss of essential metals needed by normal individuals [9]. Furthermore, research on “detoxifier” for heavy metals in relation to COPD is extremely scarce [10]. Therefore, there is an urgent need to seek practical and feasible solutions.

**Fig. 1:**
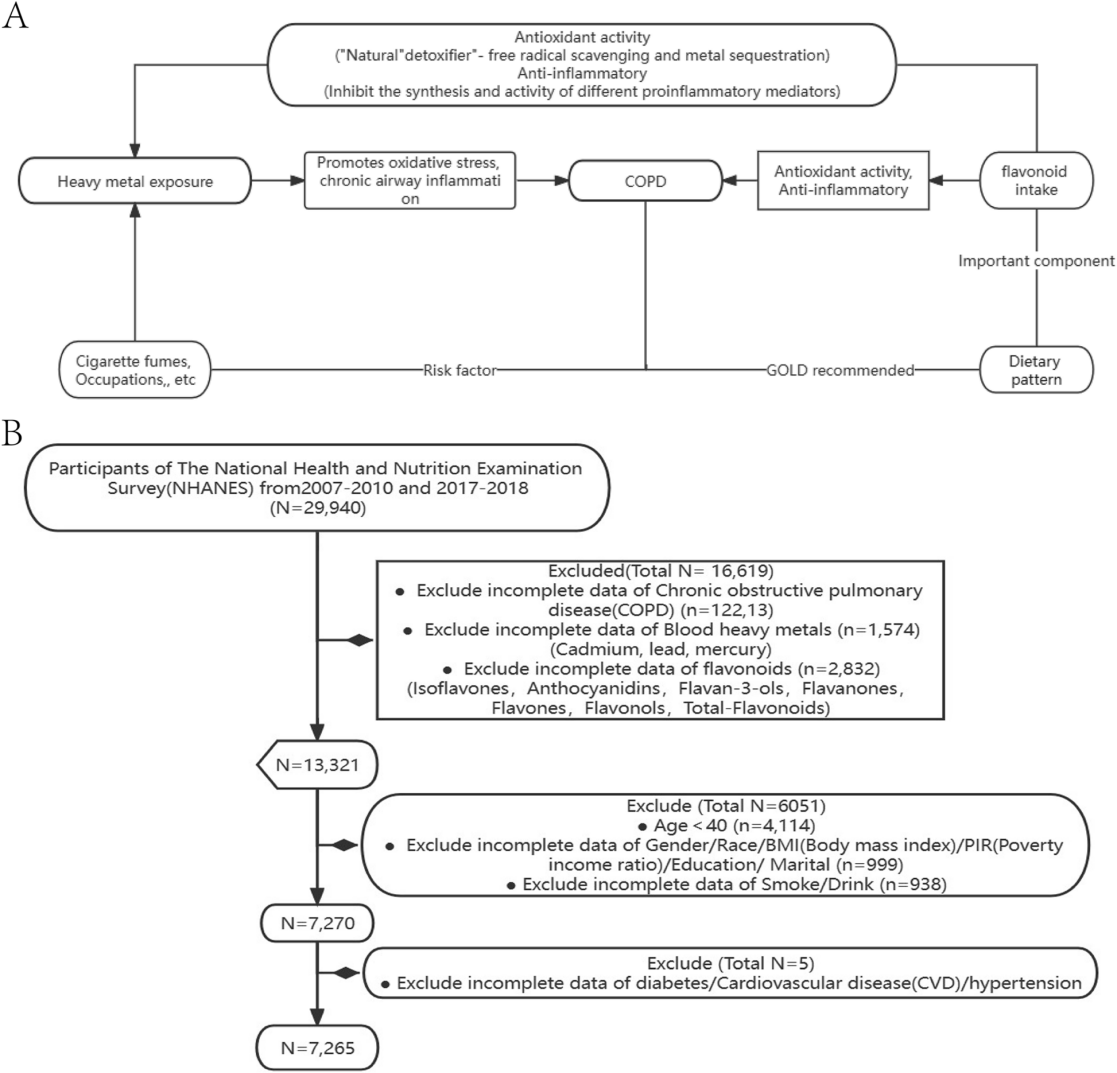
**A** The design concept of this study (GOLD: Global Initiative for Chronic Obstructive Lung Disease); **B** Inclusion flow diagram

Currently, mitigating the hazards associated with COPD-related risk factors is considered more urgent than treating COPD itself [11]. For instance, numerous studies are exploring new strategies to mitigate the increased risk of COPD due to smoking by improving dietary habits [12, 13]. Among these efforts, research on dietary polyphenols has garnered significant attention from scholars. Flavonoids represent the most important type of polyphenolic compounds, accounting for 60% of total dietary polyphenols, widely present in common fruits, vegetables, tea, soybeans, grains, and processed foods. According to their structure, they can be divided into six subcategories, including isoflavones, Anthocyanidins (AC), flavan-3-ols, flavanones, and flavonols [14]. It is known that flavonoids, which possess antioxidant and anti-inflammatory capabilities, can act as natural dietary agents and inhibitors to counteract the harmful effects of heavy metal exposure [15]. These compounds have shown associations with improved lung function indicators in COPD patients who are smokers [16, 17], and they also exhibit potential for COPD treatment [18, 19], which has been validated in animal experiments and cohort observations [20, 21]. Oxidative stress and inflammatory reactions play a crucial roles in the development of COPD, which is also the main toxic side effect of heavy metals [6, 22]. So, can the intake of flavonoid compounds serve as a natural “detoxifier” against heavy metal exposure-induced COPD? (Fig. 1A) Flavonoids have the advantages of strong availability, minimal side effects compared to traditional chelating agents, affordability, ease of acquisition, and food-medicine homology [9, 23]. These numerous advantages highlight the high feasibility of using flavonoids for COPD prevention [24]. Currently, no research has explored whether consuming flavonoids can reduce the risk of COPD development triggered by heavy metal exposure. Therefore, the significance of this study lies in exploring the impact of blood heavy metal levels/dietary flavonoid intake on the incidence of COPD through the The National Health and Nutrition Examination Survey (NHANES) database, and elucidating the potential of reducing the risk of COPD by consuming flavonoid compounds to mitigate heavy metal exposure.

## Materials and methods

### Study population

The NHANES study is a multi-stage, stratified, and nationally representative study of the US population conducted by the National Center for Health Statistics of the Centers for Disease Control and Prevention. It aims to assess the nutrition and health status of Americans [25]. The survey includes demographic, dietary, examination, laboratory, and questionnaire data. Prior to data collection, all study procedures were authorized by the ethics review committee of the National Center for Health Statistics, and informed consent forms were signed by all participants. Subjects were excluded from our study for the following reasons: (1) Missing COPD data; (2) Missing flavonoids and heavy metal data; (3) Aged under 40 years old; (4) Missing covariate (such as smoking, alcohol consumption, hypertension, diabetes, etc.) data (Fig. 1B).

### Dependent variables

Chronic obstructive pulmonary disease (COPD) was defined differently for the 2007–2010 period compared to the 2017–2018 period. In the 2007–2010 period, COPD was defined as FEV1/FVC < 0.7. However, for the 2017–2018 period, it was defined as a positive response to any of the following questions: (1) “Has a doctor or other health professional ever told you that you have chronic bronchitis?“ (2) “Has a doctor or other health professional ever told you that you have emphysema?“ (3) “Has a doctor or other health professional ever told you that you have COPD?“ (4) “Are you over 40 years old and have a history of smoking or chronic bronchitis, and take one of the following medications: mast cell stabilizers, inhaled corticosteroids, leukotriene modifiers, or selective phosphodiesterase-4 inhibitors?“

### Independent variables

In this study, flavonols intake data were obtained from the USDA’s Food and Nutrient Database for Dietary Studies (FNDDS) associated with NHANES for 2007–2010 and 2017–2018. The FNDDS provides estimates of Flavonoids intake for the US population by age group. The relationship between flavonoid intake and human health can be comprehensively explored by linking these estimates with laboratory, examination, and questionnaire data from NHANES. The flavonol database in FNDDS provides intake amounts of six major flavonols and 29 subtypes as well as total flavonols for each participant on the first and second days. In this study, we used the average intake of the six major flavonols on the first and second days for each participant in the flavonol database to investigate their association with COPD incidence.

Whole blood samples were obtained from individuals who fasted overnight at the Mobile Examination Center (MEC). All specimens were prepared, stored, and sent for analysis to the National Center for Environmental Health and the Centers for Disease Control and Prevention according to laboratory procedure manuals. Inductively coupled plasma mass spectrometry (ICP-MS) was used to assess the levels of trace elements in biological samples, and the levels of lead, Cd, and mercury in whole blood were determined [26]. Values below the limit of detection (LOD) for heavy metal concentrations were handled by dividing the LOD by the square root of 2 [27].

### Covariate assessment

Our covariates included age, sex, race/ethnicity (Mexican American, other Hispanic, non-Hispanic white, non-Hispanic black, non-Hispanic Asian, other race), BMI, smoking status, alcohol consumption status, marital status, education level (less than 9th grade, 9–11 grade, high school graduate, some college/AA degree, and college graduate), poverty income ratio (PIR), cardiovascular disease, hypertension, and diabetes. PIR was divided into three categories: Low (≤ 1.39), Medium (> 1.39, <=3.49), and High (> 3.49). Smoking status was classified into two categories: Smoker (defined as smoking more than 100 cigarettes in their lifetime), Non-smoke (defined as smoking less than 100 cigarettes in their lifetime). Alcohol consumption status was classified as lifetime abstainer (drank < 12 times in their lifetime), former drinker (drank > 12 times in their lifetime but did not drink in the past year), current light drinker (currently drinks ≤ 3 times per week), current moderate drinker (men drink > 3 to ≤ 14 times/week, women drink > 3 to ≤ 7 times/week), Current heavy drinker (men drink > 14 times/week, women drink > 7 times/week) and binge drinking (defined as “how many days in the past year did you have 5 or more drinks?“) and the raw answer was converted to days per week (or month). For CVD, a positive response to any of the following questions was defined as CVD: “Has a doctor or other health professional ever told you that you have congestive heart failure (CHF)/coronary heart disease (CHD)/angina/heart attack/stroke?“. Hypertension was defined according to the American Heart Association/American College of Cardiology 2017 guidelines as systolic blood pressure ≥ 130 mmHg or diastolic blood pressure ≥ 80 mmHg and self-reported diagnosis or use of antihypertensive medication. Diabetes was defined as meeting any of the following criteria: (1) self-reported diabetes diagnosis; (2) use of diabetes medication; (3) HbA1c > 6.5%; (4) fasting blood glucose of at least 7 mmol/L.

### Statistical analysis

All the data used in this study are publicly available and can be accessed using relevant keywords on the NHANES official website (https://wwwn.cdc.gov/nchs/nhanes/search/default.aspx). These data include serum heavy metals, personal demographic information, and potential health conditions (such as age, gender, race, education level, smoking status, and chronic underlying diseases). It is worth noting that flavonoid dietary data can be obtained from the Food and Nutrient Database for Dietary Studies (FNDDS, https://www.ars.usda.gov/northeast-area/beltsville-md-bhnrc/beltsville-human-nutrition-research-center/food-surveys-research-group/docs/fndds-download-databases/). Subsequently, the data were merged based on each individual’s unique identifier “Patient serial number (SEQN)”. Furthermore, NHANES data release files provide various sample weights, such as the MEC exam weight (wtmec2 year) [25]. Considering the complex sampling design of NHANES, sample weights were assigned to each participant to ensure that the study population is nationally representative. Since the sample individuals for the MEC exam are a subset of the survey respondents, the choice of the correct sample weight for analysis depends on the variables used. Therefore, we used the combined wtmec2 year for our analysis. NHANES 2007–2010 combines data from two survey cycles (four years), while NHANES 2017–2018 combines data from one survey cycle (two years). Consequently, different weights were applied for different years:“ wtmec2 year (2007–2010) = 2/3×wtmec2 year, wtmec2 year (2017–2018) = 1/3×wtmec2 year.

The intake of flavonoids and the exposure levels to heavy metals were divided into three quantiles (Q1, Q2, and Q3 representing the first, second, and third quantiles, respectively), with Q1 used as the reference. Continuous variables were reported as mean ± standard deviation (SD), while categorical variables were presented as individual counts (N) and percentages (%). Weighted t-tests (for continuous variables) or weighted chi-square tests (for categorical variables) were used to assess differences between COPD and non-COPD subjects. Weighted t-tests can account for variance differences between samples, allowing for a more accurate comparison of mean differences between two groups. Meanwhile, categorical variables can be assessed using weighted chi-squared tests. Weighted chi-squared tests consider weight differences between samples, thus providing a more accurate assessment of proportion differences between two groups. The Kruskal-Wallis test is a non-parametric method suitable for comparing median differences among multiple groups. For categorical variables, weighted chi-squared tests can also be employed, considering sample weight differences. Multivariable linear regression and restricted cubic splines (RCS) were used to analyze the correlation between flavonoids, heavy metals, and COPD. RCS flexibly model the relationship between independent and dependent variables, especially in regression analysis. When the relationship between independent and dependent variables is not a simple linear one, RCS can help capture this non-linear relationship. They allow researchers to approximate the relationship using different polynomial functions within different ranges of independent variables, thereby providing a more accurate description of the data’s trend. RCS with three knots, at the 10th, 50th, and 90th percentages, were used to explore the non-linear relationships of blood heavy metal levels and flavonoid intake with COPD in the linear terms model. For the construction of the logistic regression model, initially, an crude model was fitted, followed by stepwise adjustment for covariates. Model 1 adjusted for age, sex, and race; Model 2 further adjusted for PIR, BMI, marital status, education level, alcohol consumption, and smoking status based on Model 1; Model 3 additionally adjusted for cardiovascular disease, hypertension, and diabetes based on Model 2. Results were presented as odds ratios (OR) with their corresponding 95% confidence intervals (95% CI). Subgroup analyses were conducted for significant results. To further explore the association between flavonoid intake, heavy metal levels, and COPD, a logistic regression model was employed to assess the significance of the interaction between flavonoids and heavy metals on COPD. Additionally, for covariates that showed significant differences between COPD and non-COPD participants, subgroup analyses and interaction exploration were conducted to investigate their impact on the relationship between blood heavy metal levels/flavonoid intake and COPD.

All subgroup variable analyses were conducted using a fully adjusted Model 3, and logistic regression models were also used to determine the interaction associations between these subgroups and COPD.Statistical analysis was performed using R (version 4.2.2, Foundation for Statistical Computing, Vienna, Austria). All the aforementioned analysis methods were considered statistically significant with two-tailed p-values < 0.05.

## Result

### The baseline characteristics of the participants

Finally, a total of 7,265 individuals were included in this study. Based on the exclusion criteria, a total of 1,008 participants (7,265, 13%) were classified as having COPD, with a mean age of 60.2 years. Compared to non-COPD participants (6,257), those with COPD were generally older, non-Hispanic white, with higher household incomes, and were more likely to be overweight/obese (BMI ≥ 25 kg/m^2^). Besides, COPD participants were more likely to be smokers and have comorbidities like diabetes and hypertension. Among them, blood Cd (*p* < 0.001) and lead (*p* < 0.001) showed statistically significant differences between COPD and non-COPD individuals, with higher median blood levels of these heavy metals among COPD participants. In contrast, anthocyanidins (*p* = 0.013), isoflavones (*p* < 0.001), and flavones (*p* = 0.039) exhibited significantly lower median levels of intake among COPD participants (Table 1). This reflects the dietary inadequacies in COPD patients.

**Table 1:** Participant characteristics stratified by COPD status

Characteristic	Overall, *N* = 7265 (100%)^1^	No, *N* = 6257 (87%)^1^	Yes, *N* = 1008 (13%)^1^	*P*- Value^2^
Age (years) ***	57.6 (11.4)	57.2 (11.3)	60.2 (11.1)	**< 0.001**
**Body mass index (BMI)**				0.15
* Normal(< 25)*	1,701 (24%)	1,459 (25%)	242 (21%)	
* Obese(≥ 30)*	2,976 (41%)	2,536 (41%)	440 (46%)	
* Overweight(≥ 25,<30)*	2,588 (34%)	2,262 (35%)	326 (33%)	
**Sex**				0.4
* Female*	3,723 (54%)	3,232 (53%)	491 (55%)	
* Male*	3,542 (46%)	3,025 (47%)	517 (45%)	
Race***				**< 0.001**
* Non-Hispanic White*	3,702 (75%)	3,041 (74%)	661 (81%)	
* Non-Hispanic Black*	1,394 (9.2%)	1,225 (9.4%)	169 (8.5%)	
* Mexican American*	1,026 (5.9%)	965 (6.4%)	61 (2.6%)	
* Other Hispanic*	664 (4.1%)	596 (4.2%)	68 (2.9%)	
* Other Race - Including Multi-Racial*	479 (6.0%)	430 (6.0%)	49 (5.0%)	
Education***				**< 0.001**
* 9-11th grade (Includes 12th grade with no diploma)*	1,032 (9.1%)	862 (8.6%)	170 (12%)	
* College graduate or above*	1,649 (31%)	1,492 (33%)	157 (22%)	
* High school graduate/GED or equivalent*	1,711 (25%)	1,442 (25%)	269 (30%)	
* Less than 9th grade*	829 (4.4%)	711 (4.3%)	118 (5.6%)	
* Some college or AA degree*	2,044 (30%)	1,750 (30%)	294 (30%)	
**Marital**				0.2
* Divorced*	1,051 (14%)	872 (14%)	179 (16%)	
* Living with partner*	327 (4.9%)	283 (4.9%)	44 (4.7%)	
* Married*	4,315 (65%)	3,776 (66%)	539 (61%)	
* Never married*	496 (5.7%)	427 (5.6%)	69 (5.9%)	
* Separated*	242 (2.2%)	203 (2.1%)	39 (3.0%)	
* Widowed*	834 (8.1%)	696 (7.9%)	138 (9.6%)	
Poverty index (PIR)***				**< 0.001**
* High(> 3.49)*	2,548 (52%)	2,292 (54%)	256 (40%)	
* Low(≤ 1.39)*	2,119 (16%)	1,741 (15%)	378 (25%)	
* Medium(> 1.39,<=3.49)*	2,598 (32%)	2,224 (31%)	374 (35%)	
Alcohol***				**< 0.001**
* former*	1,330 (11%)	1,071 (10.0%)	259 (18%)	
* heavy*	1,043 (15%)	892 (14%)	151 (18%)	
* mild*	2,868 (47%)	2,508 (48%)	360 (40%)	
* moderate*	1,048 (18%)	900 (18%)	148 (17%)	
* never*	976 (9.8%)	886 (10%)	90 (7.4%)	
Smoking status***				**< 0.001**
* Non-smoker*	3,697 (55%)	3,451 (59%)	246 (27%)	
* smoker*	3,568 (45%)	2,806 (41%)	762 (73%)	
Diabetes***				**< 0.001**
* DM*	1,863 (20%)	1,541 (19%)	322 (30%)	
* IFG*	432 (6.6%)	376 (6.6%)	56 (6.5%)	
* IGT*	300 (2.8%)	264 (2.9%)	36 (2.8%)	
* no*	4,670 (70%)	4,076 (72%)	594 (61%)	
Hypertension***	4,896 (62%)	4,154 (61%)	742 (71%)	**< 0.001**
Cardiovascular disease (CVD)***	1,127 (12%)	842 (10%)	285 (25%)	**< 0.001**
Blood Lead***	0.015 (0.013)	0.015 (0.013)	0.018 (0.013)	**< 0.001**
Blood Cadmium***	0.49 (0.55)	0.45 (0.49)	0.77 (0.82)	**< 0.001**
**Blood Mercury**	1.62 (2.23)	1.65 (2.26)	1.45 (1.99)	0.050
Isoflavones***	1.79 (8.81)	1.92 (9.31)	0.91 (3.84)	**< 0.001**
Anthocyanidins *	17.37 (38.26)	17.99 (39.59)	13.12 (27.06)	**0.013**
**Flavan-3-ols**	195.37 (404.20)	193.63 (396.32)	207.38(454.94)	0.6
**Flavanones**	12.75 (23.53)	12.90 (23.67)	11.69(22.46)	0.4
Flavones*	1.02 (2.22)	1.04 (2.34)	0.89 (1.13)	**0.039**
**Flavonols**	19.88 (18.67)	19.89 (18.48)	19.81 (19.89)	> 0.9
**Total Flavonoids**	248.19 (422.85)	247.38 (415.08)	253.79(473.23)	0.8

*P < 0.05; **P < 0.01; ***P < 0.001

^1^Mean ± SD for continuous; n (%) for categorical

^2^t-test adapted to complex survey samples; chi-squared test with Rao & Scott's second-order correction

We analyzed the relationship between blood heavy metal levels (Cd, lead) and flavonols intake (AC, isoflavones, flavones) at the third quantile and COPD. Analysis of the third quantile of blood Cd and lead levels showed that variables such as age, gender, race, education, marital status, PIR, alcohol consumption, smoking status, diabetes, and CVD were significantly different (*p* < 0.05), this could be attributed to the potential ease with which lead exposure enters the bloodstream [28]. Additionally, the prevalence of hypertension was significantly different between the third quantile groups of blood lead levels (Supplementary Table S1, 2). The analysis of the third quantile of anthocyanidin and isoflavone intake showed that variables such as age, gender, race, education, marital status, PIR, alcohol consumption, smoking status, diabetes, hypertension, and CVD were significantly different. However, there was no significant difference in COPD prevalence in the third quantile group of flavone intake (*p* = 0.2) (Supplementary Table S3, 4 and 5).

### Associations between heavy metals, flavonols, and COPD outcomes

In the multivariable logistic regression model, participants in the third quantile of blood Cd levels in Model 3 showed a 72% increase in the risk of COPD in participants compared to the first quantile (≤ 0.24 ug/L) (OR: 1.73, 95% CI: 1.25–2.38, *p* < 0.01). Regarding blood lead levels,the third quantile was significantly positively correlated in Model 1 and Model 3. Among flavonols, the third quantile of anthocyanidin intake was statistically significant in Model 3, and compared to the first quantile (≤ 1.015 mg/d), the third quantile (≥ 11.56 mg/d) reduced the risk of COPD (OR: 0.73, 95% CI: 0.54–0.99, *p* < 0.01) (Table 2). For other models such as Mercury, Flavan-3-ols, Flavanones, Flavonols, and total Flavonoids, the association with COPD ceased to be significant after adjusting for general patient characteristics (age, gender, race). This suggests that these factors are more influenced by the environment in which patients live (education status, income level, etc.) and their underlying health conditions (such as hypertension, diabetes). We further used the RCS model to fit the nonlinear relationship between Cd, AC, and COPD, and after adjusting for covariates, we found a linear relationship between Cd (*p* < 0.001, Supplementary Figure S1A), AC (*p* < 0.001, Supplementary Figure S1B), and COPD.

**Table 2:** Associations between blood heavy metal levels (Cadmium, lead) and flavonoids intake (Anthocyanidins, Isoflavones, Flavones) with COPD in NHANES.

	Crude Model OR (95% CI)	Model 1 OR (95% CI)	Model 2 OR (95% CI)	Model3 OR (95% CI)
**Blood_Cadmium (ug/L)**				
Q1(≤ 0.24)	Reference	Reference	Reference	Reference
Q2(≥ 0.25,≤0.44)	1.19(0.87,1.62)	1.12(0.80,1.56)	0.90(0.62,1.29)	0.91(0.64,1.29)
Q3((≥ 0.45,≤3.03)	3.10(2.32,4.13) ***	3.04(2.28,4.06) ***	1.70(1.23,2.35) **	1.73(1.25,2.38) **
**Blood_Lead (ug/L)**				
Q1(< 0.105)	Reference	Reference	Reference	Reference
Q2(≥ 0.106,≤0.171)	1.42(1.05,1.92) *	1.36(1.00,1.86)	1.15(0.84,1.57)	1.25(0.94,1.66)
Q3(≥ 0.172)	1.92(1.48,2.50) ***	1.76(1.33,2.32) ***	1.30(0.98,1.73)	1.44(1.11,1.86)**
**Anthocyanidins (mg)**				
Q1(≤ 1.015)	Reference	Reference	Reference	Reference
Q2(≥ 1.02,≤11.53)	0.72(0.55,1.19)*	0.70(0.53,0.92)*	0.82(0.63,1.06)	0.85(0.65,1.11)
Q3(≥ 11.56)	0.59(0.44,0.80) ***	0.54(0.40,0.72) ***	0.72(0.53,0.96) *	0.73(0.54,0.99)*
**Isoflavones (mg)**				
Q1(<5 × 10^−3^)	Reference	Reference	Reference	Reference
Q2(≥ 5 × 10^−3^,≤0.04)	0.94(0.75,1.19)	0.93(0.74,1.18)	1.07(0.84,1.37)	1.08(0.85,1.38)
Q3(≥ 0.04)	0.71(0.56,0.90) **	0.76(0.60,0.97)*	0.93(0.71,1.22)	0.93(0.71,1.23)
**Flavones (mg)**				
Q1(≤ 0.33)	Reference	Reference	Reference	Reference
Q2(≥ 0.34,≤1.00)	0.83(0.67,1.04)	0.83(0.66,1.05)	0.98(0.79,1.22)	0.97(0.78,1.21)
Q3(≥ 1.01)	0.82(0.62,1.08)	0.82(0.61,1.10)	1.07(0.81,1.41)	1.05(0.81,1.38)
**Blood_Mercury (ug/L)**				
Q1(0.0-0.65)	Reference	Reference	Reference	Reference
Q2(0.66–1.39)	0.72(0.52, 1.00)	0.71(0.51, 0.98)*	0.82(0.59, 1.14)	0.84(0.60, 1.19)
Q3(≥ 1.40)	0.73(0.56, 0.96)*	0.70(0.52, 0.94)*	0.95(0.69, 1.31)	0.99(0.71, 1.36)
**Flavan-3-ols (mg)**				
Q1(0-10.115)	Reference	Reference	Reference	Reference
Q2(10.12-102.040)	0.79(0.64, 0.97)*	0.78(0.63, 0.95)*	0.96(0.79, 1.17)	1.01(0.82, 1.25)
Q3(≥ 102.285)	0.88(0.64, 1.21)	0.86(0.63, 1.19)	1.06(0.78, 1.46)	1.10(0.79, 1.53)
**Flavanones (mg)**				
Q1(0-1.170)	Reference	Reference	Reference	Reference
Q2(1.175–7.190)	0.87(0.71, 1.06)	0.85(0.69, 1.05)	1.08(0.86, 1.34)	1.09(0.87, 1.37)
Q3(>7.220)	0.74(0.56, 0.98)*	0.70(0.52, 0.92)*	0.90(0.66, 1.23)	0.92(0.68, 1.26)
**Flavonols (mg)**				
Q1(0-10.215)	Reference	Reference	Reference	Reference
Q2(10.225–21.405)	0.91(0.67, 1.24)	0.90(0.65, 1.23)	1.06(0.76, 1.48)	1.08(0.76, 1.54)
Q3(>21.41)	0.86(0.61, 1.21)	0.87(0.62, 1.22)	1.03(0.73, 1.45)	1.07(0.75, 1.53)
**Total Flavonoids (mg)**				
Q1(0-45.2)	Reference	Reference	Reference	Reference
Q2(45.225–179.71)	0.73(0.57, 0.93)	0.70(0.55, 0.89)**	0.95(0.74, 1.20)	0.97(0.74, 1.27)
Q3(>179.825)	0.87(0.64, 1.18)	0.84(0.61, 1.14)	1.09(0.80, 1.49)	1.11(0.81, 1.53)

Model 1: Adjusted for age, sex, and race

Model 2: Adjusted for age, sex, race, PIR (Poverty Index), BMI, marital status, education level, smoking status, and alcohol consumption

Model 3: Adjusted for age, sex, race, PIR (Poverty Index), BMI, marital status, education level, smoking status, alcohol consumption, hypertension, cardiovascular disease, and diabetes

*P < 0.05; **P < 0.01; ***P < 0.001

### Subgroup analysis

**Fig. 2:**
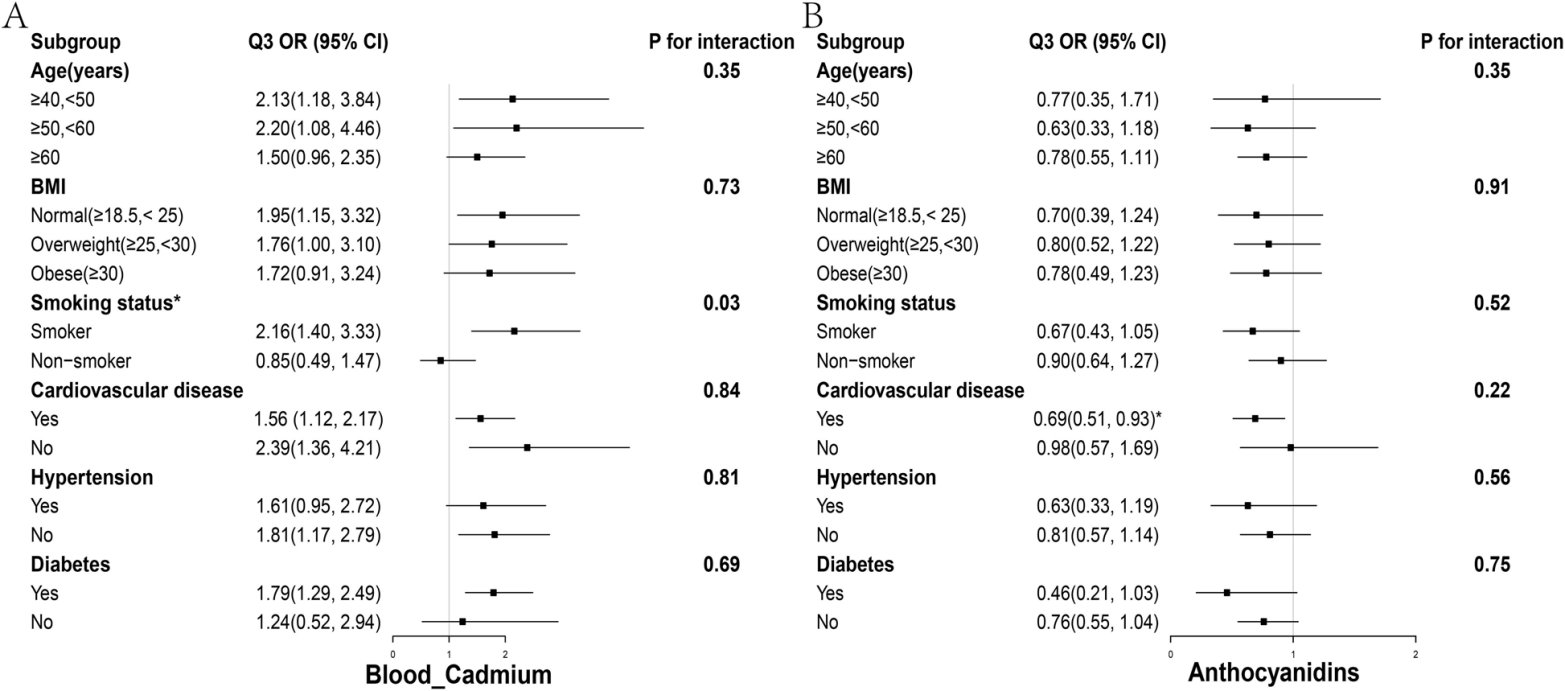
Subgroup analysis of the relationship between blood cadmium levels, anthocyanidins intake, and COPD risk at different levels

Subgroup analysis of AC and Cd showed that compared to the first quantile, the third quantile of blood Cd levels was significantly associated with a reduced risk of COPD among participants aged 40 to 60 years, with normal BMI levels, smokers, without CVD and hypertension, and with diabetes. The interaction results showed that the effect of blood Cd levels on COPD varied depending on smoking status (p for interaction = 0.03) (Fig. 2A). In terms of AC, compared to the first quantile, the second quantile of AC intake was significantly associated with a reduced risk of COPD among participants aged 50 to 60 years (Fig. 2B). For the subgroup analysis of blood lead levels and COPD, smokers, individuals with higher BMI, non-alcohol drinkers, and those with hypertension or coronary heart disease showed a higher risk of COPD onset. However, interaction analysis revealed that these factors did not have a decisive impact on the risk of COPD associated with blood lead (interaction p-values all > 0.05, Supplementary Figure S2).

To investigate whether the intake of these flavonoids can reduce the risk of heavy metal exposure-related COPD, we also conducted stratified analyses of flavonoid intake levels and blood heavy metal levels. In the additional stratified analysis of AC intake and blood Cd levels, we analyzed their combined effects on COPD incidence (Table 3). We found that participants with high blood Cd levels (> 0.44 ug/L) and inadequate AC intake (< 1.015 mg/d) had a significantly increased risk of COPD (OR: 4.88, 95% CI: 2.45–9.71, *p* < 0.001). However, participants with sufficient AC intake (> 11.53 mg/d) at the same blood Cd level experienced a 27% reduced risk of COPD (OR: 2.99, 95% CI: 1.31–4.02, *p* = 0.006). We also found an interaction between blood Cd and AC on COPD incidence (p for interaction = 0.037). Further subgroup analysis of smoking status showed this association was more significant in smokers (Table 4), these findings can provide dietary recommendations for clinical practice to reduce COPD risk associated with cadmium exposure through the consumption of AC. When we conducted stratified analysis for the remaining five types of flavonoids (Isoflavones, Flavan-3-ols, Flavanones, Flavones, Flavonols), and Total Flavonoids in relation to cadmium, we observed that at lower levels of these flavonoid compounds (at Q1 levels), the risk of COPD increased by 1.48 times, 2.27 times, 2.92 times, 1.5 times, 2.01 times, and 2.35 times, respectively, as cadmium levels rose to 0.45 µg/L. An interesting observation is that the same results were also found in the interaction analysis of flavonoids with lead (except for AC, Flavonols, and Total Flavonoids). For Isoflavones (Q1 level), Flavones (Q1 level), Flavanones (Q2 level), and Flavan-3-ols (Q2 level), the risk of COPD increased by 65%, 134%, 83%, and 72%, respectively, as cadmium levels rose to 0.172 µg/L (Supplementary Materials Tables S6, 7). However, further increasing the intake of these flavonoids did not contribute to the improvement of this risk (COPD associated with cadmium and lead exposure). This may be because increasing flavonoid intake often accompanies other dietary changes, leading to increased heterogeneity among this population. Further interaction analysis helps us conclude that, overall, there is no direct association between the intake of these flavonoid compounds and COPD risk in relation to heavy metals (blood lead and cadmium). These important findings provide clinical researchers with a direction for further investigating the potential benefits of flavonoids in reducing the adverse health effects caused by cadmium and lead. Finally, we did not find any associations between flavonoid intake, mercury exposure, and COPD risk (Supplementary Materials Table S8). This may be due to the weaker correlation between mercury exposure and COPD risk.

**Table 3:** Combined effects of Anthocyanidins intake and blood Cd levels on COPD incidence

Characteristic Blood cadmium (ug/L)	Anthocyanidins(mg) Q1(0-1.015)	Anthocyanidins(mg) Q2(1.015–11.53)	Anthocyanidins(mg) Q3(>11.56)
Q1(0.07–0.24)	Reference	Reference	Reference
Q2(0.24–0.44)	1.57(0.78,3.19)	0.42(0.23,0.79) **	1.62(0.86,3.05)
Q3(>0.44)	4.88(2.45,9.71)***	0.82(0.50,1.36)	2.29(1.31,4.02) **

In this table, the impact of increasing blood cadmium (Cd) levels on the risk of COPD is displayed with reference to blood cadmium levels in the range of 0.07-0.24ug/L, while varying the intake of anthocyanins. For example, when blood cadmium levels exceed 0.44ug/L and anthocyanin intake is greater than 11.56 mg, the risk of developing COPD is approximately 2.29 times higher compared to when blood cadmium levels are in the range of 0.07-0.24ug/L, with an equivalent anthocyanin intake (> 11.56 mg). (**P < 0.01; ***P < 0.001)

**Table 4:** Subgroup analysis of the relationship between blood cadmium levels, anthocyanidins intake, and COPD risk at different levels stratified by smoking status

**Subgroup (Smoking)**	**Characteristic**	**Anthocyanidins(mg) Q1(0-1.015)**	**Anthocyanidins(mg) Q2(1.015–11.53)**	**Anthocyanidins(mg) Q3(>11.56)**
**Blood. Cadmium (ug/L)**	Q1(0.07,0.24)	Reference	Reference	Reference
	Q2(0.24,0.44)	1.50(0.63,3.61)	0.33(0.14,0.77) *	2.23(0.84,5.91)
	Q3(>0.44)	4.67(2.19,9.99) ^***^	0.79(0.40,1.56)	3.64(1.64,8.11) **
**Subgroup (Non-smoking)**	**Characteristic**	**Anthocyanidins(mg) Q1(0-1.015)**	**Anthocyanidins(mg) Q2(1.015–11.53)**	**Anthocyanidins(mg) Q3(>11.56)**
**Blood. Cadmium (ug/L)**	Q1(0.07,0.24)	Reference	Reference	Reference
	Q2(0.24,0.44)	1.51(0.66, 3.44)	0.49(0.23, 1.07)	1.16(0.50, 2.68)
	Q3(>0.44)	1.06(0.39, 2.87)	0.60(0.22, 1.68)	1.05(0.55, 2.03)

This table further explores the impact of smoking as a factor on the interaction between blood cadmium (Cd) levels, anthocyanin intake, and COPD risk. Using a reference blood cadmium level of 0.07-0.24ug/L, while varying anthocyanin intake, it categorizes individuals based on their smoking status. For example, among former smoker individuals, when blood cadmium levels exceed 0.44ug/L and anthocyanin intake is greater than 11.56mg, the risk of developing COPD is approximately 4.36 times higher compared to when blood cadmium levels are within the range of 0.07-0.24ug/L, with an equivalent anthocyanin intake (>11.56mg). (*P < 0.05; **P < 0.01)

## Discussion

This study analyzed cross-sectional data from the NHANES project to explore the association between three blood metals (cadmium, mercury, and lead), six flavonoid compounds (isoflavones, AC, flavan-3-ols, flavanones, flavones, and flavonols), total flavonoids, and COPD-related outcomes in 7,265 adults aged ≥ 40 years. We found that increased blood Cadmium (≥ 0.45 ug/L) and Lead (≥ 0.172 ug/L) levels were positively associated with COPD susceptibility, while dietary intake of AC was negatively associated with COPD risk. Moreover, our study found that individuals with high blood Cd levels (0.45 ug/L) had a 27% reduced risk of COPD when consuming a moderate daily intake of AC (11.53 mg/d). This protective effect was particularly significant among smokers. As for whether the intake of other types of flavonoids (isoflavones, flavan-3-ols, flavanones, flavones, and flavonols) can lead to a reduction in the risk of COPD related to cadmium exposure and the potential benefits of the flavonoids involved in this study in lowering the risk of lead-induced COPD, further exploration is required in future studies.

In the current context, the focus is on human exposure to Cd and lead, which can originate from various sources. In addition to occupational exposures in industries such as mining, smelting, electroplating, nickel and lead battery production, crystal and ceramic industries [7, 29], food contamination and smoking behaviors are also significant sources of exposure to these elements [30]. Cd and lead can adversely affect lung function through various mechanisms, including oxidative stress, inflammation, cell apoptosis, and immune responses. Oxidative stress plays a pivotal role in this regard [31, 32]. Both Cd and lead can induce the production of reactive oxygen species (ROS), including superoxide anions (O^2−^) and hydrogen peroxide (H_2_O_2_). Cd can also elevate intracellular levels of calcium and iron ions, accelerating the production of free radicals, which contribute to the generation of reactive oxygen species [33]. Lead, on the other hand, can induce ROS production by inhibiting delta-aminolevulinic acid dehydratase (ALAD), leading to the accumulation of delta-aminolevulinic acid (ALA) [34]; Additionally, both Cd and lead can inhibit the activity of various antioxidant enzymes, including superoxide dismutase (SOD), catalase (CAT), and glutathione peroxidase (GPx) [35], by binding to their -SH groups with high affinity and replacing essential cofactors, resulting in decreased enzyme activity and interference with the antioxidant defense system [31]. These effects can disrupt the barrier function of lung cells, damage collagen and elastin cross-linking, accelerate collagen and elastin damage, and ultimately lead to emphysema [36, 37]. Animal experiments have shown that rats exposed to lead exhibited thinning of the interalveolar septum, reduced alveolar area, and accumulation of macrophages in the alveoli [38, 39]; Furthermore, Cd and lead may also induce the destruction of metallothionein (MT) as part of the underlying mechanisms [40]. In terms of inflammation, Cd can increase the content of alveolar macrophages and neutrophils [41, 42], and some studies suggest that certain inflammatory cytokines, such as tumor necrosis factor-alpha (TNF-α) and monocyte chemoattractant protein-1 (MCP-1), may play intermediary roles [43]. Persistent and long-term inflammation can lead to the destruction of structural cells such as airway epithelial cells, stromal cells, and parenchymal cells. Research on lead-induced lung inflammation is relatively limited, but one NHANES study indicated that lead can increase the systemic immune inflammation index (a novel index reflecting immune inflammatory status) [44], which might explain the induction of chronic persistent lung inflammation. The toxicity of cadmium and lead also manifests in terms of cell apoptosis and immune regulation, which are also mechanisms that should not be overlooked in the risk of developing COPD. Besides the accumulation of ROS, cadmium and lead may induce mitochondrial dysfunction in bronchial epithelial cells and activate the MAPK signaling pathway, contributing to the regulation of cell apoptosis [45]. Cadmium can also interfere with the cellular uptake of some essential metals, particularly zinc, leading to airway immune dysregulation [46]. Research has also found that lead exposure reduces the percentage of CD cells [47], which may also increase susceptibility to respiratory diseases. Fortunately, we have discovered the benefits of AC in reducing the risk of Cd-induced COPD, making it possible to control such risks.

AC represents a class of water-soluble flavonoids widely present in fruits and vegetables. The dietary sources of AC include foods with high levels of natural colorants, such as red and purple berries, and cabbage [48]. Organic crops with high antioxidant concentrations usually have lower levels of Cd and pesticides [49]. Extracts of AC can reduce the stress caused by Cd in the soil on crops [50]. Studies have shown that AC plays a role in preventing and treating many chronic diseases, such as metabolic disorders, cancer, and cardiovascular diseases [51, 52]. However, there is little research on the protective effects of AC in the respiratory system, especially in the prevention of COPD. A recent longitudinal study of elderly adults from the Normative Aging Study lasting more than 15 years found that the intake of AC was negatively correlated with the rate of decline in FVC [53]. Ahmad S F et al. [54] showed that feeding mice with original proanthocyanidins could improve lung inflammation by reducing pro-inflammatory cytokines produced by Th2 cells. There is a rich literature available on the protective effects of AC on Cd toxicity in humans, animals, and plants, as AC’s strong antioxidant capacity can chelate Cd^2+^ and accelerate Cd metabolism in the body, inhibit cell DNA fragmentation and apoptosis [55], and reduce the release of TNF-α, interleukin 6 (IL-6), interleukin 1 (IL-1), and numero (NO), achieving an inhibitory effect on inflammatory infiltration [15]. However, it is important to note that the above-mentioned studies have shortcomings, such as being limited to animal model studies, having small sample sizes, or lacking definitive guidance on AC intake levels. Our study found for the first time that a daily intake of 11.53 mg of AC could improve the increased risk of COPD caused by long-term Cd exposure. An interesting phenomenon is that a certain amount of AC intake can offset the risk of COPD caused by Cd exposure when blood Cd levels are in the Q2 interval (0.25–0.44 µg/L). We explain that in the population with blood Cd levels in the Q2 interval, 61% are non-smokers and 39% are smokers, significantly different from the population in the Q3 interval (Supplementary Table S1). Smoking is the most significant risk factor for COPD, and Cd is the heavy metal element with the highest cigarette content. Each cigarette produces 0.5-1 µg of Cd [56], and during smoking, Cd is deposited locally in lung tissue or absorbed into the systemic circulation, leading to a higher accumulation of Cd in the body than other heavy metals, exacerbating oxidative stress and inflammatory reactions in the body [46]. This also explains the significant difference in the incidence of COPD in this interval population, and subgroup analysis shows that AC has a more significant effect on reducing COPD risk in the population with high blood Cd levels who smoke, further supporting the viewpoint of this study. This interesting phenomenon may be related to the significant overlap between the oxidative stress and persistent inflammatory response pathways caused by smoking [57, 58] and Cd exposure and the pathways regulated by AC [15]. Thus, further exploration of the potential mechanisms is warranted.

Currently, Americans have inadequate intake of AC and are at high risk of heavy metal exposure. A previous follow-up study of up to 17,900 individuals showed that the intake of AC by American adults was 9.2 mg/d [59]. However, other studies have shown that the intake of AC by Americans aged 19 and above was only 3.1 mg/d [60]. This discrepancy may be due to heterogeneity in dietary assessment methods, age, race, and sample size. Overall, the consumption of AC in the American population falls short of the recommended intake of 11.53 mg/d as proposed in this study. Adequate supplementation of AC may be beneficial in reducing the risk of COPD, whether in the presence of cadmium exposure or in everyday life. For instance, incorporating foods such as strawberries (at a daily quantity exceeding 200 g) or common black beans (at a daily quantity of 350 g) could serve as effective means of supplementation (as outlined in the supplementary material Table S9). In addition, AC are metabolized into structurally diverse metabolites in the body and can remain in the bloodstream for up to 48 h [61, 62], indicating that AC exert beneficial effects on lung tissue through the blood circulation.

Nonetheless, the limitations of this study should be acknowledged. Firstly, a high intake of AC may represent a healthier lifestyle, and although adjustments were made for variables such as poverty index and education level, it is challenging to completely eliminate all residual or unmeasured confounding factors. However, the interaction analysis conducted between AC intake and blood Cd levels in relation to COPD minimized the impact of this problem. Secondly, it is worth noting that apart from lung cancer, there is still limited epidemiological research on flavonoids and lung health, and the cross-sectional nature of the data inherently limits our ability to establish a causal relationship between AC and reduced COPD risk. It is imperative to replicate our findings in prospective studies for validation, and further research is needed to confirm the role of AC in reducing COPD risk.

## Conclusion

When blood cadmium levels are greater than 0.45ug/L or blood lead levels are greater than 0.172ug/L, the risk of COPD increases significantly. Anthocyanidins intakes more than 11.53 mg/d significantly reduces the risk of COPD. Interaction analysis indicates that this intake can reduce the risk of COPD caused by Cd exposure by 27%. However, currently, there is no evidence to suggest that flavonoid intake can reduce the risk of lead-related COPD, and further validation of our conclusions is necessary in the future.

### Supplementary Information

**Additional file 1: Table S1.** Participant characteristics divided by Blood_Cadmium levels (NHANES 2007-2010, 2017-2018; *N* = 7,265). **Table S2.** Participant characteristics divided by Blood_Lead levels (NHANES 2007-2010, 2017-2018; *N* = 7,265). **Table S3.** Participant characteristics divided by Anthocyanidins intake levels (NHANES 2007-2010, 2017-2018; *N* = 7,265). **Table S4.** Participant characteristics divided by Isoflavones intake levels (NHANES 2007-2010, 2017-2018; *N* = 7,265). **Table S5.** Participant characteristics divided by Flavonesintake levels (NHANES 2007-2010, 2017-2018; *N* = 7,265). **Table S6.** Combined effects of Flavonoid intake and blood Cadmium levels on COPD incidence. **Table S7.** Combined effects of Flavonoid intake and blood Lead levels on COPD incidence. **Table S8.** Combined effects of Flavonoid intake and blood Mercury levels on COPD incidence. **Figure S1.** Analysis of the relationship between blood cadmium levels, anthocyanidins intake, and COPD using restricted cubic spline models. Figure represents the relationship between Cadmium, anthocyanidins intake, and COPD adjusted for age, sex, race, PIR (Poverty Index), BMI, marital status, education level, smoking status, alcohol consumption, hypertension, coronary heart disease, and diabetes.   The solid red line represents the combined restricted cubic spline curve model, and the shaded area represents the 95% confidence interval of the combined curve (There is no Log conversion for COPD variables in the figure). The dashed line represents the risk of developing COPD when blood cadmium levels (A) are in Q1 (0.07-0.24ug/L) or anthocyanin intake (B) is in Q1 (0-1.015mg). **Table S9.** Nutrient reference table for anthocyanin-rich foods. **Figure S2.** Subgroup analysis of the relationship between blood lead levels and COPD risk.

## Data Availability

Flavonoid data source: https://www.ars.usda.gov/northeast-area/beltsville-md-bhnrc/beltsville-human-nutrition-research-center/food-surveys-research-group/docs/fndds-download-databases/. Blood heavy metal data source: https://wwwn.cdc.gov/nchs/nhanes/search/default.aspx (Key words: blood cadmium, blood lead, blood mercury).
