# Supplementary material for: Heavy metal levels and flavonoid intakes are associated with chronic obstructive pulmonary disease: an NHANES analysis (2007–2010 to 2017–2018)
Source: BMC Public Health. 2023 Nov 24;23:2335. doi: 10.1186/s12889-023-17250-x (PMC10675902; doi:10.1186/s12889-023-17250-x)

Supplementary material

[1. Table S1 Participant characteristics divided by Blood_Cadmium levels (NHANES 2007-2010, 2017-2018; N = 7,265) 2](#_Toc149413087)

[2. Table S2 Participant characteristics divided by Blood_Lead levels (NHANES 2007-2010, 2017-2018; N = 7,265) 4](#_Toc149413088)

[3. Table S3 Participant characteristics divided by Anthocyanidins intake levels (NHANES 2007-2010, 2017-2018; N = 7,265). 6](#_Toc149413089)

[4. Table S4 Participant characteristics divided by Isoflavones intake levels (NHANES 2007-2010, 2017-2018; N = 7,265) 8](#_Toc149413090)

[5. Table S5 Participant characteristics divided by Flavonesintake levels (NHANES 2007-2010, 2017-2018; N = 7,265) 10](#_Toc149413091)

[6. Table S6. Combined effects of Flavonoid intake and blood Cadmium levels on COPD incidence. 12](#_Toc149413092)

[7. Table S7. Combined effects of Flavonoid intake and blood Lead levels on COPD incidence. 13](#_Toc149413093)

[8. Table S8. Combined effects of Flavonoid intake and blood Mercury levels on COPD incidence. 14](#_Toc149413094)

[9. Figure S1. Analysis of the relationship between blood cadmium levels, anthocyanidins intake, and COPD using restricted cubic spline models. 15](#_Toc149413095)

[10. Table S9. Nutrient reference table for anthocyanin-rich foods 16](#_Toc149413096)

[11. Figure S2. Subgroup analysis of the relationship between blood lead levels and COPD risk. 17](#_Toc149413097)

# Table S1 Participant characteristics divided by Blood_Cadmium levels (NHANES 2007-2010, 2017-2018; N = 7,265)

| **Characteristic** | **Overall**, N = 7265 (100%)^1^ | **Q1**, N = 1937 (34%)^1^ | **Q2**, N = 2462 (33%)^1^ | **Q3**, N = 2866 (33%)^1^ | **P Value**^2^ |
| --- | --- | --- | --- | --- | --- |
| **Age (years)***** | 57.6 (11.4) | 55.1 (10.2) | 59.1 (11.7) | 58.7 (11.6) | **<0.001** |
| **Body mass index (BMI)***** |  |  |  |  | **<0.001** |
| *Normal(<25)* | 1,701 (24%) | 316 (17%) | 516 (24%) | 869 (32%) |  |
| *Obese(≥30)* | 2,976 (41%) | 941 (49%) | 1,047 (41%) | 988 (34%) |  |
| *Overweight(≥25,<30)* | 2,588 (34%) | 680 (34%) | 899 (36%) | 1,009 (34%) |  |
| **Sex***** |  |  |  |  | **<0.001** |
| *Female* | 3,723 (54%) | 752 (38%) | 1,340 (59%) | 1,631 (64%) |  |
| *Male* | 3,542 (46%) | 1,185 (62%) | 1,122 (41%) | 1,235 (36%) |  |
| **Race***** |  |  |  |  | **<0.001** |
| *Non-Hispanic White* | 3,702 (76%) | 986 (76%) | 1,211 (77%) | 1,505 (75%) |  |
| *Non-Hispanic Black and Other Races* | 1,873 (15%) | 497 (12%) | 584 (14.3%) | 792 (16.8%) |  |
| *Hispanic* | 1,690 (9.5%) | 607 (12%) | 519 (8.7%) | 564 (8.2%) |  |
| **Education***** |  |  |  |  | **<0.001** |
| *High educational level* | 3,360 (56%) | 1,013 (60%) | 1,087 (57%) | 1,260 (51%) |  |
| *Low educational level* | 1,861 (15%) | 409 (10%) | 556 (14%) | 896 (21%) |  |
| *Medium educational level* | 2,044 (29%) | 668 (30%) | 671 (30%) | 705 (27%) |  |
| **Marital** |  |  |  |  | >0.9 |
| *Married* | 6,769 (94%) | 1,937 (94%) | 2,160 (94%) | 2,672 (94%) |  |
| *Never married* | 496 (5.8%) | 153 (5.8%) | 154 (5.8%) | 189 (5.8%) |  |
| **Poverty index (PIR)***** |  |  |  |  | **<0.001** |
| *High(>3.49)* | 2,548 (52%) | 776 (60%) | 992 (55%) | 780 (40%) |  |
| *Low(≤1.39)* | 2,119 (16%) | 461 (10%) | 610 (14%) | 1,048 (25%) |  |
| *Medium(>1.39,<=3.49)* | 2,598 (32%) | 700 (30%) | 860 (31%) | 1,038 (35%) |  |
| **Alcohol***** |  |  |  |  | **0.032** |
| *Drinker* | 6,289 (90%) | 1,756 (88%) | 1,985 (90%) | 2,548 (91%) |  |
| *Non-drinker* | 976 (10%) | 334 (12%) | 329 (10%) | 313 (8.6%) |  |
| **Smoking_status***** |  |  |  |  | **<0.001** |
| *Current Smoker* | 1,295 (16%) | 175 (7.4%) | 375 (14%) | 745 (26%) |  |
| *Former Smoker* | 2,273 (30%) | 538 (25%) | 711 (30%) | 1,024 (36%) |  |
| *Never-Smoker* | 3,697 (54%) | 1,377 (67%) | 1,228 (56%) | 1,092 (38%) |  |
| **Diabetes*** |  |  |  |  | **0.039** |
| *Diabetes mellitus(DM)* | 1,863 (20%) | 538 (21%) | 660 (21%) | 665 (18%) |  |
| *Impaired fasting glucose(IFG)* | 432 (6.6%) | 118 (6.7%) | 140 (6.3%) | 174 (6.9%) |  |
| *Impaired glucose tolerance(IGT)* | 300 (2.8%) | 64 (1.7%) | 117 (3.7%) | 119 (3.2%) |  |
| *no* | 4,670 (70%) | 1,217 (70%) | 1,545 (69%) | 1,908 (72%) |  |
| **Hypertension** | 4,896 (62%) | 1,258 (60%) | 1,673 (62%) | 1,965 (63%) | 0.6 |
| **Coronary artery disease(CVD)***** | 1,127 (12%) | 232 (9.9%) | 342 (11%) | 553 (15%) | **0.017** |
| **Chronic obstructive pulmonary disease(COPD)** |  |  |  |  | **<0.001** |
| *COPD* | 1,008 (13%) | 165 (7.9%) | 246 (9.3%) | 597 (21%) |  |
| *Non-COPD* | 6,257 (87%) | 1,772 (92%) | 2,216 (91%) | 2,269 (79%) |  |

^1^Mean ± SD for continuous; n (%) for categorical.

^2^t-test adapted to complex survey samples; chi-squared test with Rao & Scott's second-order correction.

# Table S2 Participant characteristics divided by Blood_Lead levels (NHANES 2007-2010, 2017-2018; N = 7,265)

| **Characteristic** | **Overall**, N = 7265 (100%)^1^ | **Q1**, N = 1805 (34%)^1^ | **Q2**, N = 2318 (33%)^1^ | **Q3**, N = 3142 (33%)^1^ | **P Value**^2^ |
| --- | --- | --- | --- | --- | --- |
| **Age (years)***** | 57.6 (11.4) | 54.0 (10.8) | 57.9 (10.8) | 61.0 (11.3) | **<0.001** |
| **Body mass index (BMI)***** |  |  |  |  | **<0.001** |
| *Normal(<25)* | 1,701 (24%) | 347 (20%) | 470 (21%) | 884 (31%) |  |
| *Obese(≥30)* | 2,976 (41%) | 926 (50%) | 1,013 (43%) | 1,037 (31%) |  |
| *Overweight(≥25,<30)* | 2,588 (34%) | 532 (30%) | 835 (36%) | 1,221 (38%) |  |
| **Sex***** |  |  |  |  | **<0.001** |
| *Female* | 3,723 (54%) | 1,204 (64%) | 1,249 (52%) | 1,270 (44%) |  |
| *Male* | 3,542 (46%) | 601 (36%) | 1,069 (48%) | 1,872 (56%) |  |
| **Race**** |  |  |  |  | **0.002** |
| *Mexican American* | 3,702 (76%) | 986 (76%) | 1,211 (77%) | 1,505 (75%) |  |
| *Other Hispanic* | 1,873 (15%) | 497 (12%) | 584 (14.3%) | 792 (16.8%) |  |
| *Other Race - Including Multi-Racial* | 1,690 (9.5%) | 607 (12%) | 519 (8.7%) | 564 (8.2%) |  |
| **Education***** | 3,360 (56%) | 1,013 (60%) | 1,087 (57%) | 1,260 (51%) | **<0.001** |
| *High educational level* | 1,861 (15%) | 409 (10%) | 556 (14%) | 896 (21%) |  |
| *Low educational level* | 2,044 (29%) | 668 (30%) | 671 (30%) | 705 (27%) |  |
| *Medium educational level* |  |  |  |  |  |
| **Marital**** | 6,769 (94%) | 1,937 (94%) | 2,160 (94%) | 2,672 (94%) | **0.008** |
| *Married* | 496 (5.8%) | 153 (5.8%) | 154 (5.8%) | 189 (5.8%) |  |
| *Never married* | 3,360 (56%) | 1,013 (60%) | 1,087 (57%) | 1,260 (51%) |  |
| **Poverty index (PIR)***** |  |  |  |  | **<0.001** |
| *High(>3.49)* | 2,548 (52%) | 707 (59%) | 862 (50%) | 979 (46%) |  |
| *Low(≤1.39)* | 2,119 (16%) | 459 (13%) | 625 (16%) | 1,035 (20%) |  |
| *Medium(>1.39,<=3.49)* | 2,598 (32%) | 639 (29%) | 831 (34%) | 1,128 (33%) |  |
| **Alcohol***** |  |  |  |  | **<0.001** |
| *Drinker* | 6,289 (90%) | 1,756 (88%) | 1,985 (90%) | 2,548 (91%) |  |
| *Non-drinker* | 976 (10%) | 334 (12%) | 329 (10%) | 313 (8.6%) |  |
| **Smoking_status***** |  |  |  |  | **<0.001** |
| *Current Smoker* | 1,295 (16%) | 175 (7.4%) | 375 (14%) | 745 (26%) |  |
| *Former Smoker* | 2,273 (30%) | 538 (25%) | 711 (30%) | 1,024 (36%) |  |
| *Never-Smoker* | 3,697 (54%) | 1,377 (67%) | 1,228 (56%) | 1,092 (38%) |  |
| **Diabetes**** |  |  |  |  | **0.002** |
| *Diabetes mellitus(DM)* | 1,863 (20%) | 568 (25%) | 593 (19%) | 702 (16%) |  |
| *Impaired fasting glucose(IFG)* | 432 (6.6%) | 104 (6.8%) | 125 (5.6%) | 203 (7.5%) |  |
| *Impaired glucose tolerance(IGT)* | 300 (2.8%) | 55 (1.8%) | 103 (3.1%) | 142 (3.6%) |  |
| *no* | 4,670 (70%) | 1,078 (66%) | 1,497 (72%) | 2,095 (73%) |  |
| **Hypertension*** | 4,896 (62%) | 1,123 (59%) | 1,562 (61%) | 2,211 (66%) | **0.025** |
| **Coronary artery disease(CVD)***** | 1,127 (12%) | 182 (8.7%) | 341 (13%) | 604 (15%) | **<0.001** |
| **Chronic obstructive pulmonary disease(COPD)***** |  |  |  |  | **<0.001** |
| *COPD* | 1,008 (13%) | 189 (9.2%) | 306 (13%) | 513 (16%) |  |
| *Non-COPD* | 6,257 (87%) | 1,616 (91%) | 2,012 (87%) | 2,629 (84%) |  |

^1^Mean ± SD for continuous; n (%) for categorical.

^2^t-test adapted to complex survey samples; chi-squared test with Rao & Scott's second-order correction.

# Table S3 Participant characteristics divided by Anthocyanidins intake levels (NHANES 2007-2010, 2017-2018; N = 7,265).

| **Characteristic** | **Overall**, N = 7265 (100%)^1^ | **Q1**, N = 2560 (33%)^1^ | **Q2**, N = 2710 (33%)^1^ | **Q3**, N = 1995 (33%)^1^ | **P Value**^2^ |
| --- | --- | --- | --- | --- | --- |
| **Age (years)***** | 57.6 (11.4) | 56.1 (11.2) | 58.0 (11.3) | 58.7 (11.3) | **<0.001** |
| **Body mass index (BMI)***** |  |  |  |  | **<0.001** |
| *Normal(<25)* | 1,701 (24%) | 577 (21%) | 572 (22%) | 552 (29%) |  |
| *Obese(≥30)* | 2,976 (41%) | 1,150 (48%) | 1,138 (43%) | 688 (34%) |  |
| *Overweight(≥25,<30)* | 2,588 (34%) | 833 (31%) | 1,000 (35%) | 755 (37%) |  |
| **Sex*** |  |  |  |  | **0.029** |
| *Female* | 3,723 (54%) | 1,229 (50%) | 1,390 (53%) | 1,104 (57%) |  |
| *Male* | 3,542 (46%) | 1,331 (50%) | 1,320 (47%) | 891 (43%) |  |
| **Race***** |  |  |  |  | **<0.001** |
| *Mexican American* | 3,702 (76%) | 1,264 (73%) | 1,181 (72%) | 1,257 (83%) |  |
| *Other Hispanic* | 1,873 (15%) | 805 (18.2%) | 630 (15%) | 438 (10.3%) |  |
| *Other Race - Including Multi-Racial* | 1,690 (9.5%) | 531 (8.8%) | 802 (13%) | 357 (6.7%) |  |
| **Education***** |  |  |  |  | **<0.001** |
| *High educational level* | 3,360 (56%) | 1,085 (50%) | 1,146 (55%) | 1,129 (62%) |  |
| *Low educational level* | 1,861 (15%) | 816 (20%) | 760 (17%) | 285 (8.7%) |  |
| *Medium educational level* | 2,044 (29%) | 699 (30%) | 707 (28%) | 638 (29%) |  |
| **Marital**** |  |  |  |  | **0.028** |
| *Married* | 6,769 (94%) | 2,379 (93%) | 2,461 (95%) | 1,929 (95%) |  |
| *Never married* | 496 (5.8%) | 221 (7.3%) | 152 (4.7%) | 123 (5.4%) |  |
| **Poverty index (PIR)***** |  |  |  |  | **<0.001** |
| *High(>3.49)* | 2,548 (52%) | 696 (43%) | 852 (49%) | 1,000 (64%) |  |
| *Low(≤1.39)* | 2,119 (16%) | 931 (23%) | 830 (17%) | 358 (9.0%) |  |
| *Medium(>1.39,<=3.49)* | 2,598 (32%) | 933 (35%) | 1,028 (34%) | 637 (27%) |  |
| **Alcohol***** |  |  |  |  | **<0.001** |
| *Drinker* | 6,289 (90%) | 2,259 (91%) | 2,219 (87%) | 1,811 (91%) |  |
| *Non-drinker* | 976 (10%) | 341 (9.3%) | 394 (13%) | 241 (8.9%) |  |
| **Smoking_status***** |  |  |  |  | **<0.001** |
| *Current Smoker* | 1,295 (16%) | 697 (24%) | 393 (14%) | 205 (9.1%) |  |
| *Former Smoker* | 2,273 (30%) | 746 (29%) | 836 (30%) | 691 (33%) |  |
| *Never-Smoker* | 3,697 (54%) | 1,157 (47%) | 1,384 (56%) | 1,156 (58%) |  |
| **Diabetes*** |  |  |  |  | **0.038** |
| *Diabetes mellitus(DM)* | 1,863 (20%) | 693 (22%) | 752 (21%) | 418 (17%) |  |
| *Impaired fasting glucose(IFG)* | 432 (6.6%) | 166 (7.7%) | 156 (5.7%) | 110 (6.5%) |  |
| *Impaired glucose tolerance(IGT)* | 300 (2.8%) | 100 (2.6%) | 115 (3.1%) | 85 (2.9%) |  |
| *no* | 4,670 (70%) | 1,601 (68%) | 1,687 (70%) | 1,382 (74%) |  |
| **Hypertension***** | 4,896 (62%) | 1,779 (67%) | 1,825 (61%) | 1,292 (57%) | **<0.001** |
| **CVD** | 1,127 (12%) | 448 (14%) | 396 (12%) | 283 (11%) | 0.2 |
| **Chronic obstructive pulmonary disease(COPD)** |  |  |  |  | **0.002** |
| *COPD* | 1,008 (13%) | 436 (16%) | 346 (12%) | 226 (10%) |  |
| *Non-COPD* | 6,257 (87%) | 2,124 (84%) | 2,364 (88%) | 1,769 (90%) |  |

^1^Mean ± SD for continuous; n (%) for categorical.

^2^t-test adapted to complex survey samples; chi-squared test with Rao & Scott's second-order correction.

# Table S4 Participant characteristics divided by Isoflavones intake levels (NHANES 2007-2010, 2017-2018; N = 7,265)

| **Characteristic** | **Overall**, N = 7265 (100%)^1^ | **Q1**, N = 2560 (33%)^1^ | **Q2**, N = 2710 (33%)^1^ | **Q3**, N = 1995 (33%)^1^ | **P Value**^2^ |
| --- | --- | --- | --- | --- | --- |
| **Age (years) ***** | 57.6 (11.4) | 56.1 (11.2) | 58.0 (11.3) | 58.7 (11.3) | **<0.001** |
| **Body mass index (BMI)***** |  |  |  |  | **<0.001** |
| *Normal(<25)* | 1,701 (24%) | 316 (17%) | 516 (24%) | 869 (32%) |  |
| *Obese(≥30)* | 2,976 (41%) | 941 (49%) | 1,047 (41%) | 988 (34%) |  |
| *Overweight(≥25,<30)* | 2,588 (34%) | 680 (34%) | 899 (36%) | 1,009 (34%) |  |
| **Sex*** |  |  |  |  | **0.029** |
| *Female* | 3,723 (54%) | 1,229 (50%) | 1,390 (53%) | 1,104 (57%) |  |
| *Male* | 3,542 (46%) | 1,331 (50%) | 1,320 (47%) | 891 (43%) |  |
| **Race***** |  |  |  |  | **<0.001** |
| *Mexican American* | 3,702 (76%) | 1,493 (77%) | 1,188 (79%) | 1,021 (72%) |  |
| *Other Hispanic* | 1,873 (15%) | 740 (15%) | 527 (12.3%) | 606 (16%) |  |
| *Other Race - Including Multi-Racial* | 1,690 (9.5%) | 514 (8.0%) | 487 (8.7%) | 689 (12%) |  |
| **Education***** |  |  |  |  | **<0.001** |
| *High educational level* | 3,360 (56%) | 1,221 (54%) | 1,036 (56%) | 1,103 (58%) |  |
| *Low educational level* | 1,861 (15%) | 748 (18%) | 544 (14%) | 569 (13%) |  |
| *Medium educational level* | 2,044 (29%) | 778 (29%) | 622 (29%) | 644 (29%) |  |
| **Marital**** |  |  |  |  | **0.028** |
| *Married* | 6,769 (94%) | 2,556 (94%) | 2,063 (95%) | 2,150 (94%) |  |
| *Never married* | 496 (5.8%) | 191 (6.4%) | 139 (5.1%) | 166 (5.6%) |  |
| **Poverty index (PIR)***** |  |  |  |  | **<0.001** |
| *High(>3.49)* | 2,548 (52%) | 696 (43%) | 852 (49%) | 1,000 (64%) |  |
| *Low(≤1.39)* | 2,119 (16%) | 931 (23%) | 830 (17%) | 358 (9.0%) |  |
| *Medium(>1.39,<=3.49)* | 2,598 (32%) | 933 (35%) | 1,028 (34%) | 637 (27%) |  |
| **Alcohol***** |  |  |  |  | **<0.001** |
| *Drinker* | 6,289 (90%) | 2,363 (88%) | 1,904 (91%) | 2,022 (91%) |  |
| *Non-drinker* | 976 (10%) | 384 (12%) | 298 (9.3%) | 294 (9.3%) |  |
| **Smoking_status***** |  |  |  |  | **<0.001** |
| *Current Smoker* | 1,295 (16%) | 566 (19%) | 372 (15%) | 357 (13%) |  |
| *Former Smoker* | 2,273 (30%) | 881 (32%) | 681 (30%) | 711 (29%) |  |
| *Never-Smoker* | 3,697 (54%) | 1,300 (49%) | 1,149 (55%) | 1,248 (59%) |  |
| **Diabetes*** |  |  |  |  | **0.038** |
| *Diabetes mellitus(DM)* | 1,863 (20%) | 693 (22%) | 752 (21%) | 418 (17%) |  |
| *Impaired fasting glucose(IFG)* | 432 (6.6%) | 166 (7.7%) | 156 (5.7%) | 110 (6.5%) |  |
| *Impaired glucose tolerance(IGT)* | 300 (2.8%) | 100 (2.6%) | 115 (3.1%) | 85 (2.9%) |  |
| *no* | 4,670 (70%) | 1,601 (68%) | 1,687 (70%) | 1,382 (74%) |  |
| **Hypertension***** | 4,896 (62%) | 1,779 (67%) | 1,825 (61%) | 1,292 (57%) | **<0.001** |
| **Coronary artery disease(CVD)** | 1,127 (12%) | 448 (14%) | 396 (12%) | 283 (11%) | 0.2 |
| **Chronic obstructive pulmonary disease(COPD)**** |  |  |  |  | **0.002** |
| *COPD* | 1,008 (13%) | 436 (16%) | 346 (12%) | 226 (10%) |  |
| *Non-COPD* | 6,257 (87%) | 2,124 (84%) | 2,364 (88%) | 1,769 (90%) |  |

^1^Mean ± SD for continuous; n (%) for categorical.

^2^t-test adapted to complex survey samples; chi-squared test with Rao & Scott's second-order correction.

# Table S5 Participant characteristics divided by Flavonesintake levels (NHANES 2007-2010, 2017-2018; N = 7,265)

| **Characteristic** | **Overall**, N = 7265 (100%)^1^ | **Q1**, N = 2587 (33%)^1^ | **Q2**, N = 2529 (34%)^1^ | **Q3**, N = 2149 (33%)^1^ | **P Value**^2^ |
| --- | --- | --- | --- | --- | --- |
| **Age (years)** | 57.6 (11.4) | 57.4 (11.6) | 57.7 (11.5) | 57.8 (10.9) | 0.5 |
| **Body mass index (BMI)*** |  |  |  |  | **0.017** |
| *Normal(<25)* | 1,701 (24%) | 604 (23%) | 566 (23%) | 531 (26%) |  |
| *Obese(≥30)* | 2,976 (41%) | 1,113 (46%) | 1,018 (40%) | 845 (39%) |  |
| *Overweight(≥25,<30)* | 2,588 (34%) | 870 (32%) | 945 (37%) | 773 (35%) |  |
| **Sex***** |  |  |  |  | **<0.001** |
| *Female* | 3,723 (54%) | 1,316 (55%) | 1,351 (58%) | 1,056 (48%) |  |
| *Male* | 3,542 (46%) | 1,271 (45%) | 1,178 (42%) | 1,093 (52%) |  |
| **Race***** |  |  |  |  | **<0.001** |
| *Mexican American* | 3,702 (76%) | 1,302 (74%) | 1,241 (76%) | 1,159 (78%) |  |
| *Other Hispanic* | 1,873 (15%) | 793 (17.2%) | 582 (13%) | 498 (12.9%) |  |
| *Other Race - Including Multi-Racial* | 1,690 (9.5%) | 550 (8.8%) | 624 (11%) | 516 (9.1%) |  |
| **Education***** |  |  |  |  | **<0.001** |
| *High educational level* | 3,360 (56%) | 1,076 (50%) | 1,141 (55%) | 1,143 (62%) |  |
| *Low educational level* | 1,861 (15%) | 848 (20%) | 590 (14%) | 423 (11%) |  |
| *Medium educational level* | 2,044 (29%) | 721 (30%) | 716 (30%) | 607 (27%) |  |
| **Marital**** |  |  |  |  | **0.004** |
| *Divorced* | 6,769 (94%) | 2,424 (93%) | 2,297 (95%) | 2,048 (95%) |  |
| *Living with partner* | 496 (5.8%) | 221 (7.3%) | 150 (5.3%) | 125 (4.8%) |  |
| **Poverty index (PIR)***** |  |  |  |  | **<0.001** |
| *High(>3.49)* | 2,548 (52%) | 652 (41%) | 915 (53%) | 981 (61%) |  |
| *Low(≤1.39)* | 2,119 (16%) | 965 (22%) | 699 (16%) | 455 (11%) |  |
| *Medium(>1.39,<=3.49)* | 2,598 (32%) | 970 (36%) | 915 (31%) | 713 (28%) |  |
| **Alcohol***** |  |  |  |  | **<0.001** |
| *Drinker* | 1,048 (18%) | 369 (17%) | 371 (19%) | 308 (18%) |  |
| *Non-drinker* | 976 (9.8%) | 387 (11%) | 322 (9.4%) | 267 (9.1%) |  |
| **Smoking_status***** |  |  |  |  | **0.002** |
| *Current Smoker* | 3,697 (55%) | 1,199 (50%) | 1,314 (58%) | 1,184 (58%) |  |
| *Former Smoker* |  |  |  |  |  |
| *Never-Smoker* | 3,568 (45%) | 1,388 (50%) | 1,215 (42%) | 965 (42%) |  |
| **Diabetes** |  |  |  |  | 0.4 |
| *Diabetes mellitus(DM)* | 1,863 (20%) | 717 (22%) | 652 (19%) | 494 (19%) |  |
| *Impaired fasting glucose(IFG)* | 432 (6.6%) | 138 (5.7%) | 151 (7.4%) | 143 (6.8%) |  |
| *Impaired glucose tolerance(IGT)* | 300 (2.8%) | 105 (2.8%) | 106 (2.9%) | 89 (2.9%) |  |
| *no* | 4,670 (70%) | 1,627 (70%) | 1,620 (71%) | 1,423 (71%) |  |
| **Hypertension** | 4,896 (62%) | 1,818 (65%) | 1,693 (62%) | 1,385 (59%) | 0.074 |
| **Coronary artery disease(CVD)** | 1,127 (12%) | 432 (13%) | 415 (12%) | 280 (12%) | 0.7 |
| **COPD** |  |  |  |  | 0.2 |
| *COPD* | 1,008 (13%) | 417 (14%) | 327 (12%) | 264 (12%) |  |
| *Non-COPD* | 6,257 (87%) | 2,170 (86%) | 2,202 (88%) | 1,885 (88%) |  |

^1^Mean ± SD for continuous; n (%) for categorical.

^2^t-test adapted to complex survey samples; chi-squared test with Rao & Scott's second-order correction.

# Table S6. Combined effects of Flavonoid intake and blood Cadmium levels on COPD incidence.

| **Characteristic**  **Blood Cadmium levels (ug/L)** | **Isoflavones** (mg)  Q1(0-0.005) | **Isoflavones** (mg)  Q2(0.005-0.04) | **Isoflavones** (mg)  Q3(＞0.04) | **P for interaction** |
| --- | --- | --- | --- | --- |
| Q1(0.07-0.24) | Reference | Reference | Reference | P=0.62 |
| Q2(≥0.24,≤0.44) | 1.24(0.74, 2.08) | 0.7(0.38, 1.28) | 0.95(0.52, 1.74) |  |
| Q3(≥0.45,≤3.03) | 2.48(1.54, 3.98)*** | 1.57(0.92, 2.68) | 1.71(0.97, 3.03) |  |
| Characteristic | **Flavan-3-ols** (mg)  Q1(0-10.115) | **Flavan-3-ols** (mg)  Q2(10.12-102.040) | **Flavan-3-ols** (mg)  Q3(≥102.285) |  |
| Q1(0.07-0.24) | Reference | Reference | Reference | P=0.38 |
| Q2(≥0.24,≤0.44) | 0.89(0.46, 1.74) | 1.03(0.61, 1.74) | 0.98(0.49, 1.98) |  |
| Q3(≥0.45,≤3.03) | 3.27(1.94, 5.51)*** | 1.10(0.60, 2.03) | 1.49(0.78, 2.86) |  |
| Characteristic | **Flavanones** (mg)  Q1(0-1.170) | **Flavanones** (mg)  Q2(1.175-7.190) | **Flavanones** (mg)  Q3(＞7.220) |  |
| Q1(0.07-0.24) | Reference | Reference | Reference | P=0.43 |
| Q2(≥0.24,≤0.44) | 1.13(0.61, 2.08) | 0.77(0.42, 1.42) | 1.0(0.59, 1.68) |  |
| Q3(≥0.45,≤3.03) | 3.92(1.97, 7.81)*** | 1.06(0.55, 2.06) | 1.22(0.73, 2.04) |  |
| Characteristic | **Flavones** (mg)  Q1(0-0.330) | **Flavones** (mg)  Q2(0.335-1.000) | **Flavones** (mg)  Q3(＞1.005) |  |
| Q1(0.07-0.24) | Reference | Reference | Reference | P=0.23 |
| Q2(≥0.24,≤0.44) | 0.73(0.38, 1.41) | 1.19(0.60, 2.35) | 0.85(0.47, 1.55) |  |
| Q3(≥0.45,≤3.03) | 2.50(1.51, 4.14)*** | 2.59(0.92, 5.09) | 1.03(0.58, 1.80) |  |
| Characteristic | **Flavonols** (mg)  Q1(0-10.215) | **Flavonols** (mg)  Q2(10.225-21.405) | **Flavonols** (mg)  Q3(＞21.41) |  |
| Q1(0.07-0.24) | Reference | Reference | Reference | P=0.38 |
| Q2(≥0.24,≤0.44) | 0.81(0.46, 1.43) | 0.85(0.47, 1.53) | 1.03(0.56, 1.88) |  |
| Q3(≥0.45,≤3.03) | 3.01(1.77, 5.12)*** | 1.24(0.61, 2.52) | 1.50(0.86, 2.62) |  |
| Characteristic | **Total Flavonoids** (mg)  Q1(0-45.2) | **Total Flavonoids** (mg)  Q2(45.225-179.71) | **Total Flavonoids** (mg)  Q3(＞179.825) |  |
| Q1(0.07-0.24) | Reference | Reference | Reference | P=0.34 |
| Q2(≥0.24,≤0.44) | 0.95(0.54, 1.66) | 0.96(0.53, 1.76) | 0.93(0.47, 1.82) |  |
| Q3(≥0.45,≤3.03) | 3.35(2.07, 5.43)*** | 1.07(0.58, 1.99) | 1.46(0.79, 2.69) |  |

In this table, the impact of increasing blood cadmium (Cd) levels on the risk of COPD is displayed with reference to blood cadmium levels in the range of 0.07-0.24ug/L, while varying the intake of flavonoids. For example, when blood cadmium levels exceed 0.44ug/L and flavanones intake is less than 1.17 mg, the risk of developing COPD is approximately 2.92 times higher compared to when blood cadmium levels are in the range of 0.07-0.24ug/L, with an equivalent flavanones intake (＜1.17 mg). (***P < 0.001). Additionally, multiple linear regression was employed for interaction analysis to explore the mutual influence between overall blood cadmium levels and flavonoid intake.

# Table S7. Combined effects of Flavonoid intake and blood Lead levels on COPD incidence.

| **Characteristic**  **Blood Lead levels (ug/L)** | **Anthocyanidins (AC) (mg)**  **Q1**(0-1.015) | **Anthocyanidins(mg)**  **Q2**(1.015-11.53) | **Anthocyanidins(mg)**  **Q3**(＞11.56) | **P for interaction** |
| --- | --- | --- | --- | --- |
| Q1(<0.105) | Reference | Reference | Reference | P=0.87 |
| Q2(≥0.106,≤0.171) | 0.99(0.65,1.51) | 1.18(0.65,2.12) | 1.58(0.72,3.49) |  |
| Q3(≥0.172) | 1.04(0.73,1.47) | 1.32(0.76,2.29) | 1.79(0.93,3.42) |  |
|  | **Isoflavones** (mg)  Q1(0-0.005) | **Isoflavones** (mg)  Q2(0.005-0.04) | **Isoflavones** (mg)  Q3(＞0.04) |  |
| Q1(<0.105) | Reference | Reference | Reference | P=0.57 |
| Q2(≥0.106,≤0.171) | 1.63(1.01, 2.64)* | 0.99(0.56, 1.76) | 1.2(0.62, 2.34) |  |
| Q3(≥0.172) | 1.65(1.08, 2.52)* | 1.21(0.72, 2.03) | 1.59(0.86, 2.95) |  |
|  | **Flavan-3-ols** (mg)  Q1(0-10.115) | **Flavan-3-ols** (mg)  Q2(10.12-102.040) | **Flavan-3-ols** (mg)  Q3(≥102.285) |  |
| Q1(<0.105) | Reference | Reference | Reference | P=0.36 |
| Q2(≥0.106,≤0.171) | 1.09(0.69, 1.75) | 2.2(1.15, 4.23)* | 0.95(0.55, 1.61) |  |
| Q3(≥0.172) | 1.41(0.89, 2.22) | 2.34(1.31, 3.72)* | 1.38(0.85, 2.25) |  |
|  | **Flavanones** (mg)  Q1(0-1.170) | **Flavanones** (mg)  Q2(1.175-7.190) | **Flavanones** (mg)  Q3(＞7.220) |  |
| Q1(<0.105) | Reference | Reference | Reference | P=0.14 |
| Q2(≥0.106,≤0.171) | 1.12(0.71, 1.76) | 1.34(0.90, 2.01) | 1.45(0.80, 2.62) |  |
| Q3(≥0.172) | 1.35(0.97, 1.87) | 1.83(1.25, 2.67)** | 1.27(0.69, 2.36) |  |
|  | **Flavones** (mg)  Q1(0-0.330) | **Flavones** (mg)  Q2(0.335-1.000) | **Flavones** (mg)  Q3(＞1.005) |  |
| Q1(<0.105) | Reference | Reference | Reference | P=0.58 |
| Q2(≥0.106,≤0.171) | 1.42(0.99, 2.03) | 0.91(0.57, 1.45) | 1.65(0.78, 3.48) |  |
| Q3(≥0.172) | 1.72(1.10, 2.70)* | 1.02(0.64, 1.65) | 2.04(0.92, 4.53) |  |
|  | **Flavonols** (mg)  Q1(0-10.215) | **Flavonols** (mg)  Q2(10.225-21.405) | **Flavonols** (mg)  Q3(＞21.41) |  |
| Q1(<0.105) | Reference | Reference | Reference | P=0.53 |
| Q2(≥0.106,≤0.171) | 1.5(0.99, 2.28) | 1.42(0.84, 2.41) | 0.86(0.44, 1.69) |  |
| Q3(≥0.172) | 1.49(0.94, 2.37) | 1.43(0.78, 2.62) | 1.49(0.76, 2.93) |  |
|  | **Total Flavonoids** (mg)  Q1(0-45.2) | **Total Flavonoids** (mg)  Q2(45.225-179.71) | **Total Flavonoids** (mg)  Q3(＞179.825) |  |
| Q1(<0.105) | Reference | Reference | Reference | P=0.67 |
| Q2(≥0.106,≤0.171) | 1.27(0.83, 1.93) | 1.65(0.82, 3.33) | 1.0(0.58, 1.71) |  |
| Q3(≥0.172) | 1.5(0.96, 2.34) | 1.54(0.77, 3.09) | 1.5(0.95, 2.38) |  |

In this table, the impact of increasing blood lead levels on the risk of COPD is displayed with reference to blood lead levels less than 0.105ug/L, while varying the intake of flavonoids. For example, when blood lead levels exceed 0.172ug/L and flavanones intake are in the range of 1.175-7.19 mg/d, the risk of developing COPD is approximately 1.83 times higher compared to when blood lead levels less than 0.105ug/L, with an equivalent flavanones intake (1.175-7.19mg). (*P < 0.05; **P < 0.01). Additionally, multiple linear regression was employed for interaction analysis to explore the mutual influence between overall blood lead levels and flavonoid intake.

# Table S8. Combined effects of Flavonoid intake and blood Mercury levels on COPD incidence.

| **Characteristic**  **Blood Mercury levels (ug/L)** | **Anthocyanidins (AC) (mg)**  **Q1**(0-1.015) | **Anthocyanidins(mg)**  **Q2**(1.015-11.53) | **Anthocyanidins(mg)**  **Q3**(＞11.56) | **P for interaction** |
| --- | --- | --- | --- | --- |
| Q1(<0.65) | Reference | Reference | Reference | P=0.69 |
| Q2((≥0.66,≤1.39) | 0.84(0.55, 1.26) | 1.15(0.73, 1.79) | 0.63(0.37, 1.06) |  |
| Q3(≥1.40) | 0.77(0.43, 1.38) | 1.58(0.99, 2.44) | 0.86(0.46, 1.62) |  |
| Characteristic | **Isoflavones** (mg)  Q1(0-0.005) | **Isoflavones** (mg)  Q2(0.005-0.04) | **Isoflavones** (mg)  Q3(＞0.04) |  |
| Q1(<0.65) | Reference | Reference | Reference | P=0.25 |
| Q2((≥0.66,≤1.39) | 0.76(0.48, 1.19) | 0.74(0.41, 1.33) | 0.94(0.62, 1.44) |  |
| Q3(≥1.40) | 0.77(0.47, 1.27) | 0.96(0.55, 1.67) | 1.2(0.69, 2.10) |  |
| Characteristic | **Flavan-3-ols** (mg)  Q1(0-10.115) | **Flavan-3-ols** (mg)  Q2(10.12-102.040) | **Flavan-3-ols** (mg)  Q3(≥102.285) |  |
| Q1(<0.65) | Reference | Reference | Reference | P=0.47 |
| Q2((≥0.66,≤1.39) | 0.97(0.60, 1.56) | 0.75(0.46, 1.22) | 0.81(0.50, 1.33) |  |
| Q3(≥1.40) | 0.8(0.50, 1.30) | 1.02(0.67, 1.54) | 1.12(0.65, 1.93) |  |
| Characteristic | **Flavanones** (mg)  Q1(0-1.170) | **Flavanones** (mg)  Q2(1.175-7.190) | **Flavanones** (mg)  Q3(＞7.220) |  |
| Q1(<0.65) | Reference | Reference | Reference | P=0.24 |
| Q2((≥0.66,≤1.39) | 0.82(0.46, 1.48) | 1.19(0.77, 1.83) | 0.65(0.38, 1.09) |  |
| Q3(≥1.40) | 0.94(0.48, 1.83) | 1.40(0.92, 2.13) | 0.67(0.43, 1.05) |  |
| Characteristic | **Flavones** (mg)  Q1(0-0.330) | **Flavones** (mg)  Q2(0.335-1.000) | **Flavones** (mg)  Q3(＞1.005) |  |
| Q1(<0.65) | Reference | Reference | Reference | P=0.73 |
| Q2((≥0.66,≤1.39) | 0.71(0.47, 1.09) | 0.80(0.40, 1.62) | 0.91(0.50, 1.67) |  |
| Q3(≥1.40) | 0.84(0.50, 1.40) | 0.87(0.49, 1.56) | 1.07(0.70, 1.64) |  |
| Characteristic | **Flavonols** (mg)  Q1(0-10.215) | **Flavonols** (mg)  Q2(10.225-21.405) | **Flavonols** (mg)  Q3(＞21.41) |  |
| Q1(<0.65) | Reference | Reference | Reference | P=0.42 |
| Q2((≥0.66,≤1.39) | 1.5(0.99, 2.28) | 1.42(0.84, 2.41) | 0.86(0.44, 1.69) |  |
| Q3(≥1.40) | 1.49(0.94, 2.37) | 1.43(0.78, 2.62) | 1.49(0.76, 2.93) |  |
| Characteristic | **Total Flavonoids** (mg)  Q1(0-45.2) | **Total Flavonoids** (mg)  Q2(45.225-179.71) | **Total Flavonoids** (mg)  Q3(＞179.825) |  |
| Q1(<0.65) | Reference | Reference | Reference | P=0.72 |
| Q2((≥0.66,≤1.39) | 1.05(0.71, 1.56) | 0.59(0.32, 1.09) | 0.87(0.55, 1.39) |  |
| Q3(≥1.40) | 1.09(0.78, 1.51) | 0.72(0.42, 1.24) | 1.17(0.67, 2.05) |  |

In this table, the impact of increasing blood mercury levels on the risk of COPD is displayed with reference to blood mercury levels less than 0.65ug/L, while varying the intake of flavonoids. This table shows that regardless of the level of flavonoid intake, blood mercury levels are consistently not associated with COPD. Additionally, multiple linear regression was employed for interaction analysis to explore the mutual influence between overall blood mercury levels and flavonoid intake.

# Figure S1. Analysis of the relationship between blood cadmium levels, anthocyanidins intake, and COPD using restricted cubic spline models.


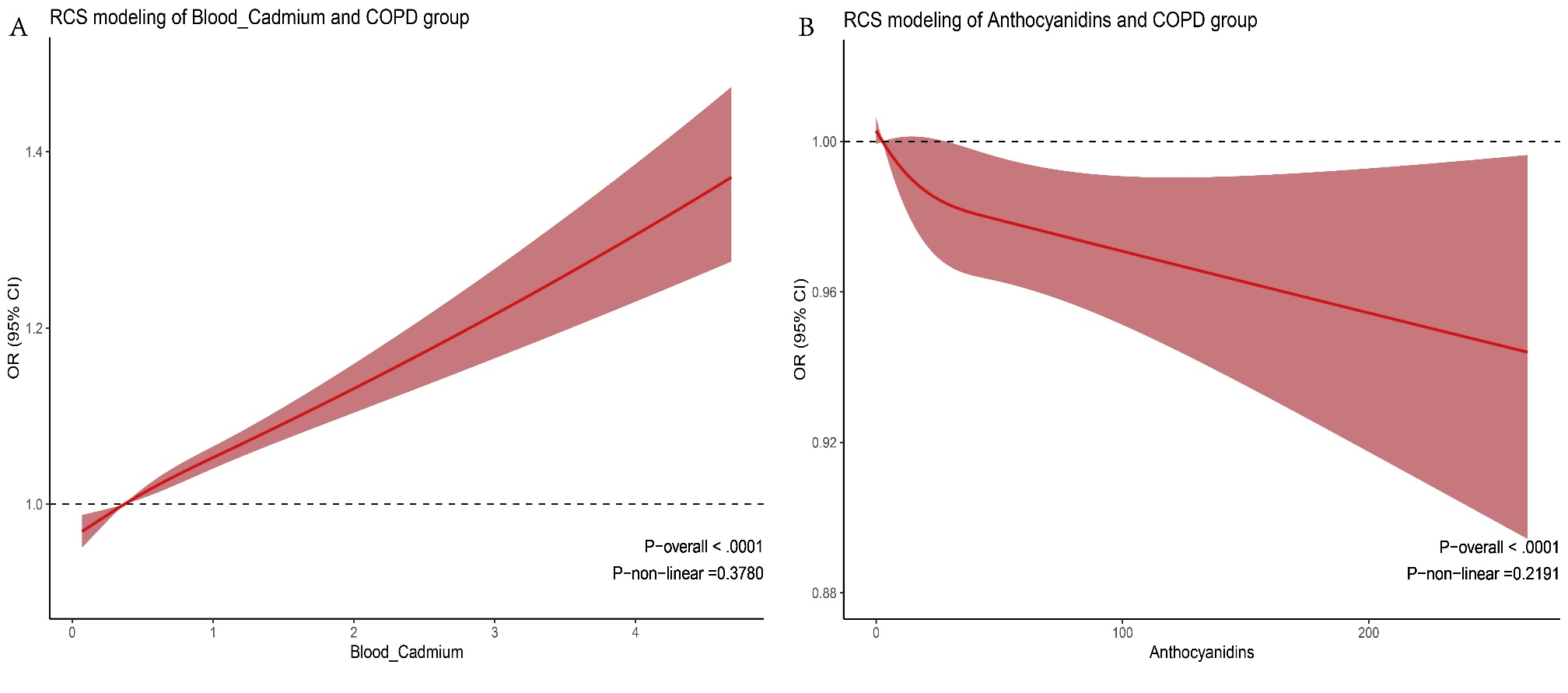
 Figure represents the relationship between Cadmium, anthocyanidins intake, and COPD adjusted for age, sex, race, PIR (Poverty Index), BMI, marital status, education level, smoking status, alcohol consumption, hypertension, coronary heart disease, and diabetes. The solid red line represents the combined restricted cubic spline curve model, and the shaded area represents the 95% confidence interval of the combined curve (There is no Log conversion for COPD variables in the figure). The dashed line represents the risk of developing COPD when blood cadmium levels (A) are in Q1 (0.07-0.24ug/L) or anthocyanin intake (B) is in Q1 (0-1.015mg).

# Table S9. Nutrient reference table for anthocyanin-rich foods

| **Main Sources** | **Latin Name of Plant Sources** | **Anthocyanidins_Class** | **Mean Content (mg/100 g)** |
| --- | --- | --- | --- |
| **Tea (Kenyan purple leaf)** | Camellia sinensis | Delphinidin | 0.12 |
|  |  | Cyanidin | 1.75 |
|  |  | Pelargonidin | 0.84 |
|  |  | Malvidin | 0.30 |
| **Common bean (black)** | Phaseolus vulgaris | Peonidin | 1.36 |
|  |  | Cyanidin | 0.53 |
|  |  | Pelargonidin | 0.95 |
| **Strawberry** | Fragaria sp | Cyanidin | 0.50 |
|  |  | Pelargonidin | 4.31 |
| **Red raspberry** | Rubus idaeus | Cyanidin | 0.53 |

# Figure S2. Subgroup analysis of the relationship between blood lead levels and COPD risk.


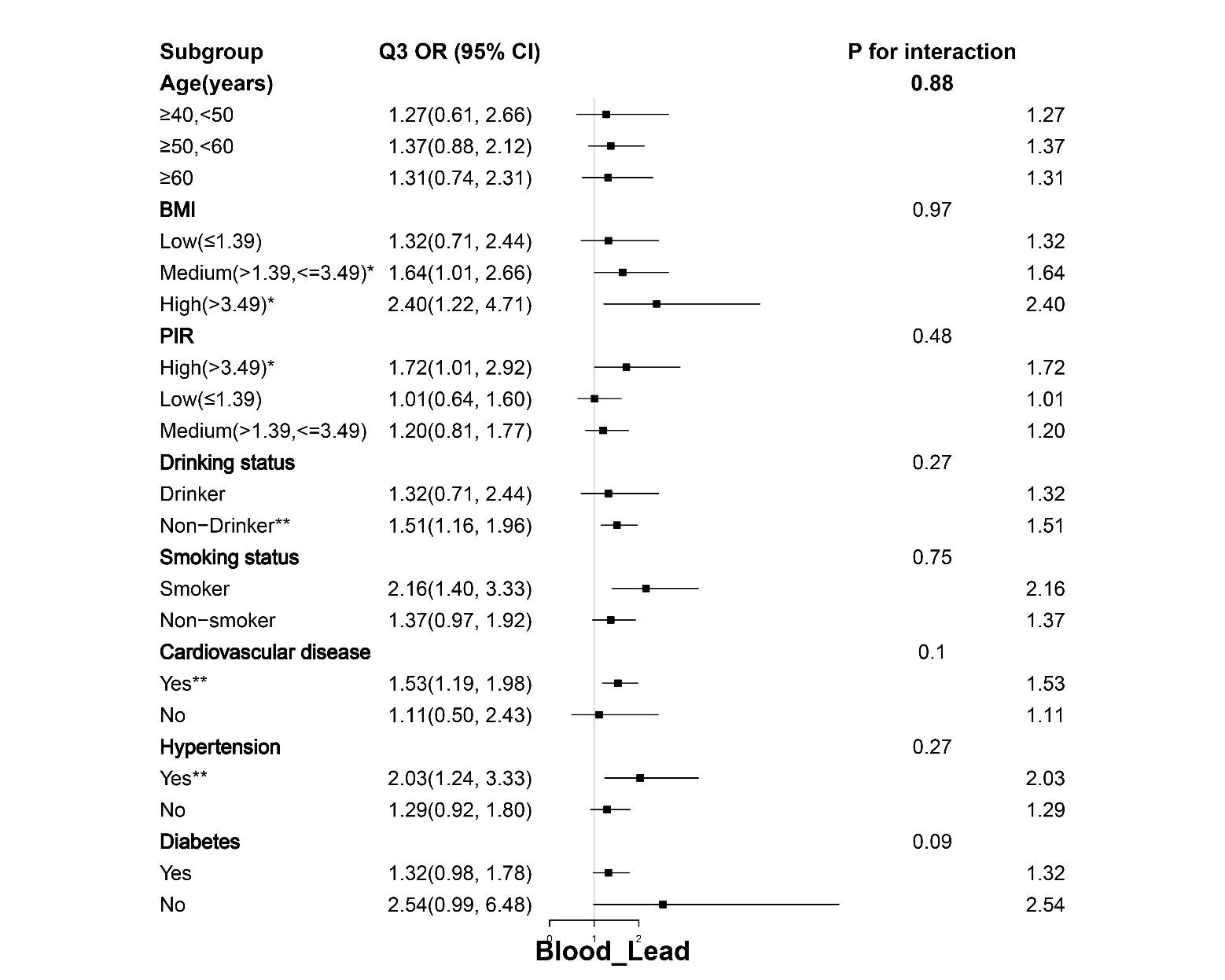

Supplement: Supplementary file 1 — Additional file 1: Table S1. Participant characteristics divided by Blood_Cadmium levels (NHANES 2007-2010, 2017-2018; N = 7,265). Table S2. Participant characteristics divided by Blood_Lead levels (NHANES 2007-2010, 2017-2018; N = 7,265). Table S3. Participant characteristics divided by Anthocyanidins intake levels (NHANES 2007-2010, 2017-2018; N = 7,265). Table S4. Participant characteristics divided by Isoflavones intake levels (NHANES 2007-2010, 2017-2018; N = 7,265). Table S5. Participant characteristics divided by Flavonesintake levels (NHANES 2007-2010, 2017-2018; N = 7,265). Table S6. Combined effects of Flavonoid intake and blood Cadmium levels on COPD incidence. Table S7. Combined effects of Flavonoid intake and blood Lead levels on COPD incidence. Table S8. Combined effects of Flavonoid intake and blood Mercury levels on COPD incidence. Figure S1. Analysis of the relationship between blood cadmium levels, anthocyanidins intake, and COPD using restricted cubic spline models. Figure represents the relationship between Cadmium, anthocyanidins intake, and COPD adjusted for age, sex, race, PIR (Poverty Index), BMI, marital status, education level, smoking status, alcohol consumption, hypertension, coronary heart disease, and diabetes. The solid red line represents the combined restricted cubic spline curve model, and the shaded area represents the 95% confidence interval of the combined curve (There is no Log conversion for COPD variables in the figure). The dashed line represents the risk of developing COPD when blood cadmium levels (A) are in Q1 (0.07-0.24ug/L) or anthocyanin intake (B) is in Q1 (0-1.015mg). Table S9. Nutrient reference table for anthocyanin-rich foods. Figure S2. Subgroup analysis of the relationship between blood lead levels and COPD risk. [file 12889_2023_17250_MOESM1_ESM.docx]
